# Determinants and Phenotypes of Poorly Controlled COPD Using the RADAR Score: A Cohort in Real-World Primary Care

**DOI:** 10.3390/jcm15031283

**Published:** 2026-02-05

**Authors:** Myriam Calle Rubio, Soha Esmaili, Juan Luis Rodríguez Hermosa, Imán Esmaili, María Carmen Antón Sanz, Norma Doria Carlin, Elías Ekech Mesa, Mónica González Álvarez, Patricia Privado Martínez, Alberto Serrano López De Las Hazas, José Artica García, María Teresa Marín Becerra, Rafael Sánchez-del Hoyo, Medardo Montenegro

**Affiliations:** 1 Pulmonology Department, Hospital Clínico San Carlos, 28040 Madrid, Spain; mcallerubio@gmail.com (M.C.R.); md.montenegrov@gmail.com (M.M.); 2 Instituto de Investigación Sanitaria del Hospital Clínico San Carlos (IdISSC), 28040 Madrid, Spain; soha@esmaili.ws; 3 Department of Medicine, School of Medicine, Universidad Complutense de Madrid, 28040 Madrid, Spain; 4 CIBER de Enfermedades Respiratorias (CIBERES), 28040 Madrid, Spain; 5 Pulmonology Department, Hospital Universitario La Zarzuela and Hospital Quirónsalud San Jose, 28023 Madrid, Spain; 6 Heart Lung Innovation Centre, Vancouver, BC V6Z 1Y6, Canada; 7 School of Medicine, Universidad Antonio de Nebrija, 28248 Madrid, Spain; 8 ISNS Data Analytics and Research, Vancouver, BC V6Z 1Y6, Canada; 9 Primary Care Center Villalba Estación, SERMAS, 28222 Madrid, Spain; mcantonsanz@gmail.com; 10 Primary Care Center Los Cármenes, SERMAS, 28047 Madrid, Spain; 11 Primary Care Center Espronceda, SERMAS, 28003 Madrid, Spain; 12 Primary Care Center Puerta Del Ángel, SERMAS, 28011 Madrid, Spain; 13 Primary Care Center Primero de Mayo, SERMAS, 28521 Madrid, Spain; 14 Primary Care Center Almodóvar, SERMAS, 28031 Madrid, Spain; 15 Primary Care Center Sector III, SERMAS, 28905 Madrid, Spain; 16 Primary Care Center General Ricardos, SERMAS, 28019 Madrid, Spain; 17 Research Methodological Support Unit and Preventive Department, Hospital Clínico San Carlos, IdISSC, 28040 Madrid, Spain

**Keywords:** chronic obstructive pulmonary disease, clinical control, therapeutic inertia, phenotype, cluster analysis, treatment adherence

## Abstract

**Background**: Poor clinical control in Chronic Obstructive Pulmonary Disease (COPD) is prevalent, yet the interplay of disease severity, modifiable factors, and clinician perception remains poorly understood. This study aimed to determine the frequency of poor control, identify its independent determinants, and characterize the heterogeneity of the poorly controlled population receiving maintenance inhaled therapy with various devices in primary care. **Methods**: In a multicenter, cross-sectional analysis of 988 patients from the Study SIMPLIFY, clinical control of COPD was classified using the objective RADAR score. We used multivariable logistic regression and Machine Learning (Random Forest with SHAP analysis) to identify determinants of poor control (RADAR ≥ 4) and k-medoids cluster analysis to characterize the poorly controlled subgroup (*n* = 452). **Results**: Nearly half the cohort (45.7%, *n* = 452) had poor clinical control. Agreement between physician-assessed control (five categories) and RADAR classification was 49.3%, with overestimation in 34.0% and underestimation in 16.7% of cases (Cohen’s κ = −0.081; weighted κ = −0.037). The strongest independent determinants were the exacerbator phenotypes (eosinophilic aOR 6.85; non-eosinophilic aOR 4.91). Key modifiable factors included active smoking (aOR 1.92), lower TAI-12 adherence score (per point; aOR 0.96), high dosing frequency (≥4 inhalations/day; aOR 1.54) and high inhaler burden (≥3 devices; aOR 1.84). Machine learning analysis identified clinical phenotype and adherence behavior as the top two scale-independent predictors of poor control. Cluster analysis of the poorly controlled group revealed five reproducible and clinically meaningful phenotypes (C0–C4), primarily separated by treatment complexity, comorbidities, and adherence. **Conclusions**: Poor clinical control is common and critically under-recognized in primary care patients with COPD on maintenance inhaled therapy. This is driven by a profound clinician perception gap and a failure to address key modifiable determinants, such as high dosing frequency, regimen complexity, and poor adherence, which likely drives therapeutic inertia. Our findings underscore the need to integrate objective tools to unmask poor control and highlight the importance of treatment simplification. The identification of distinct clinical phenotypes provides a roadmap toward a more personalized, evidence-based standard of care.

## 1. Introduction

Chronic Obstructive Pulmonary Disease (COPD) represents a global health crisis, ranking as the third leading cause of death worldwide [1] and imposing a substantial and escalating socioeconomic burden on healthcare systems [2]. Evidence-based management of patients with COPD according to recommendations of clinical practice guidelines is crucial for decreasing the impact of the disease and risk. Achieving clinical control of the disease is the overall goal in the therapeutic approach.

The concept of COPD control is a measure proposed in the Spanish COPD Guidelines (GesEPOC) based on two components—clinical impact (degree of dyspnoea, use of rescue medication, limitations in daily physical activity) and stability (absence of exacerbations in the last 3 months)—that aims to assist clinicians in assessing the clinical status of COPD patients during visits [3]. This is similar to the GOLD guidelines, which recommend that two key treatable characteristics, dyspnoea and the occurrence of exacerbations, should be assessed at each visit during treatment monitoring [4]. Evidence has shown that the COPD control status proposed by GesEPOC is a good predictor of short- and medium-term negative outcomes [5,6,7]. Recently, a scoring system for the criteria defining clinical control proposed by GesEPOC has been developed that provides a quantitative assessment, the RADAR score, which allows greater discrimination between levels of control and more direct comparisons [8,9]. This score provides a more concrete and comparable measure, facilitating a more dynamic strategy in therapeutic decisions and enabling greater personalization of treatment.

Assessing clinical control in patients with COPD is key to bridging the “perception gap,” in which physicians often fail to recognize the true extent of poor control, leading to therapeutic inertia, i.e., the failure to change therapy when treatment goals are not met, either by changing medication or increasing dosage, which has been recognized as a major factor contributing to the gap between guidelines and the appropriate treatment of COPD patients [10,11,12]. This is particularly important in high-risk populations with inhaled polypharmacy, where it is necessary to reassess modifiable determinants that we can act upon.

Among these modifiable factors, therapeutic non-adherence and critical errors in inhalation technique are a central and pervasive challenge. Adherence rates to long-term therapies in COPD average a mere 50% in developed countries [13] and are consistently linked to worse clinical outcomes, including increased hospitalization and mortality [14]. This adherence is often compromised by the complexity of the treatment regimen. Inhaler polypharmacy—the use of multiple devices—is associated not only with lower adherence [15] but also with a significantly higher risk of critical errors in inhalation technique [16], which are directly correlated with poor symptom control and an elevated risk of exacerbations [17,18]. This interplay suggests a potential “vicious cycle”: severe disease (non-modifiable) prompts the prescription of complex regimens (modifiable), which can impair adherence and technique, thus perpetuating a state of poor control. Testing the components of this hypothesis is a central aim of the current study. In recognition of this interplay, recent clinical practice guidelines increasingly advocate for treatment simplification.

The factors leading to poor control in patients in the context of routine clinical practice in primary care are not yet fully understood. There is a critical need to differentiate the relative contributions of non-modifiable disease characteristics, such as the severity of airflow limitation or an intrinsic exacerbator phenotype, from potentially modifiable factors that represent viable targets for intervention.

Accordingly, the aims of this study were:

1. To determine the distribution of the degree of good, insufficient, and poor clinical control, as classified by the RADAR score, within a large, real-world cohort of patients with COPD undergoing primary care using various devices as maintenance therapy for COPD.
2. To characterize and compare the sociodemographic, clinical, and therapeutic profiles of patients across these distinct control strata.
3. To identify the independent determinants of poor clinical control, with a specific focus on disentangling the relative contributions of non-modifiable disease severity markers and modifiable treatment-related factors.

## 2. Materials and Methods

### 2.1. Study Design

The SIMPLIFY study was an observational, multicenter, and prospective investigation conducted across primary care settings in Spain. This paper reports the cross-sectional analysis of the baseline data collected between 1 November 2023 and 1 December 2024. Participating physicians, selected for their experience in COPD research, consecutively recruited the first five eligible patients with a confirmed diagnosis of COPD who attended a routine clinical visit and met all inclusion criteria. Data were collected during a single study visit and supplemented by a review of electronic health records from the preceding year. The complete list of participating investigators is provided in the Section S2.

### 2.2. Study Population and Selection Criteria

Patients who met the following criteria were included in this study: age ≥40 years, have a spirometry-confirmed diagnosis of COPD defined by a post-bronchodilator forced expiratory volume in 1 s (FEV_1_) to forced vital capacity (FVC) ratio of <0.7, a lifetime smoking history of at least 10 pack-years and undergoing inhaled maintenance treatment using various devices

A total of 1065 patients with COPD were initially screened for eligibility, of whom 1039 met all the inclusion criteria. The final analytical cohort for this study comprised 988 patients. To maximize statistical power and minimize selection bias, missing data in covariates (4.9% of cases) were handled using multiple imputation, allowing the inclusion of all 988 eligible patients in the final multivariable regression models. The flow of patients through the study is detailed in Supplementary Figure S1.

### 2.3. Outcome Definition and Assessment

The primary outcome of this analysis was the level of clinical control, which was objectively and quantitatively assessed using the RADAR score [8]. The composite RADAR score, with a range from 0 to 8, is calculated from four key clinical domains: three points are assigned for the use of rescue medication three or more times per week; two points for experiencing one or more moderate or severe exacerbations within the last three months; two points for a dyspnea level of ≥2 on the modified Medical Research Council (mMRC) scale; and one point for self-reported walking of less than 30 min per day. In accordance with prior validation to predict future adverse events [9], patients were stratified based on their total score into three clinically distinct categories: ‘Good Control’ (score of 0–1), ‘Insufficient Control’ (score of 2–3), and ‘Poor Control’ (score of ≥4) as detailed in Section S1 (Table S4).

### 2.4. Predictor Variables and Data Collection

A comprehensive set of predictor variables was collected for each participant using a standardized case report form, with direct patient assessment and questionnaires during the study visit and supplemented by data extracted from electronic health records. Variables were selected based on established clinical relevance in COPD control literature [3,4]. Comorbidity burden was quantified using the Charlson Comorbidity Index (calculated as the weighted sum of 19 comorbid conditions), and specific individual comorbidities (e.g., asthma, heart failure) were also recorded independent of the index [19]. The severity of airflow limitation was assessed for post-bronchodilator FEV_1_ percent predicted value. Based on the prior year’s exacerbation history and blood eosinophil counts, patients were assigned a clinical phenotype according to GesEPOC as non-exacerbator, exacerbator with an eosinophilic profile, or exacerbator with a non-eosinophilic profile [3]. To ensure methodological rigor, key treatment-related variables were operationally defined to quantify the two main drivers of non-adherence: dosing frequency and number of devices [13]. Accordingly, two dichotomous variables were established for the analysis: high dosing frequency, defined as a total of four or more inhalations per day, and high inhaler burden, defined as the use of three or more inhaler devices for maintenance therapy. Finally, two critical patient-related factors were assessed: therapeutic adherence was measured using the validated Test of Adherence to Inhalers (TAI) [20], using both its categorical classification (good, intermediate, or poor) and the numerical 12-item total score (TAI-12); and the correctness of inhalation technique was evaluated by identifying the presence of any critical errors during device use. The treating physician’s subjective assessment of the patient’s overall COPD control was also recorded at the visit. The treating physician’s perceived degree of COPD control was evaluated using a 5-point Likert scale (from −2: very poor to +2: Excellent) [21]. For analytical purposes, this scale was subsequently categorized into five levels: Very Poor (scores −2), Poor (score −1), Fair (score 0), Good (score +1) and Excellent (score +2).

### 2.5. Statistical Analysis

All statistical analyses were performed using SPSS Statistics for Windows, Version 29.0 (IBM Corp., Armonk, NY, USA), and a two-sided *p*-value of less than 0.05 was considered statistically significant for all tests. An initial descriptive analysis was conducted to summarize the baseline characteristics of the cohort. Normality of continuous variables was assessed using the Shapiro–Wilk test; they were expressed as means and standard deviations (SD) if normally distributed, or medians and interquartile ranges (IQR) otherwise. Categorical variables were presented as absolute frequencies and percentages. For bivariate comparisons across the three clinical control groups, the Pearson Chi-square test (or Fisher’s exact test for expected cell counts <5) was used for categorical variables. For continuous variables, the Analysis of Variance (ANOVA) or the non-parametric Kruskal–Wallis test was used, based on the results of normality testing.

To identify the independent determinants of poor clinical control, a multivariable binary logistic regression model was developed. The dependent variable was dichotomized as ‘Poor Control’ (RADAR score ≥ 4) versus ‘Not Poor Control’ (RADAR score 0–3). To avoid collinearity and circular reasoning, variables that are components of the RADAR score itself (i.e., exacerbations in the past three months and the mMRC dyspnea score) were excluded a priori from consideration as predictors. Independent variables were considered for inclusion in the multivariable model if they demonstrated established clinical relevance and a *p*-value < 0.10 in bivariate analyses. The model’s performance was assessed by its discrimination (Area Under the Curve, AUC) and calibration (Hosmer–Lemeshow test). To visually assess the agreement between predicted probabilities and observed outcomes, calibration plots were generated. Furthermore, Clinical Utility was evaluated using Decision Curve Analysis (DCA) to quantify the net benefit of the model across a range of threshold probabilities. Given the presence of missing data in some covariates, a secondary analysis using multiple imputation (m = 20 datasets) was conducted to evaluate the robustness of the findings (see Section S1 Table S5 for comparison of original and imputed data). Imputation was performed using the Fully Conditional Specification (FCS) method with Predictive Mean Matching (PMM) for continuous variables and Logistic Regression for categorical variables (m = 20 iterations). The same set of predictors retained in the stepwise model was refitted across imputed datasets, and pooled estimates were obtained using Rubin’s rules. To further minimize model-selection bias and assess the stability of associations, a cross-validated LASSO logistic regression followed by a refit of the selected predictors was performed as an additional sensitivity analysis. A comprehensive validation of the cohort and analysis was performed; details regarding statistical power and missing data analysis are provided in the Section S1 (Section S1B), while a full diagnostic validation of the model, including for multicollinearity, is provided in the Section S1 (Section S1C). Results are presented as adjusted odds ratios (aOR) with 95% confidence intervals (CI).

To assess the agreement between the physician’s subjective rating and the objective RADAR classification, Cohen’s kappa coefficient was calculated. Finally, to identify clinically meaningful patient phenotypes within the poorly controlled COPD population (RADAR score ≥4), we conducted an unsupervised cluster analysis. The Partitioning Around Medoids (PAM) algorithm was applied to a Gower distance matrix, which accommodates mixed variable types. Nine prespecified clinical and treatment features were used: (1) Charlson comorbidity index; (2) pack-years; (3) TAI-12 adherence score; (4) number of inhaler devices (2, 3, ≥4); (5) total daily inhalations (2–3, 4–6, >6); (6) FEV_1_ severity category (≥80%, 50–79%, <50%); (7) smoking status (smoker/ex-smoker vs. never); (8) maintenance therapy with three drug classes (yes/no); and (9) inhaled corticosteroid (ICS) use (yes/no). Continuous variables were standardized (z-scores), ordinal variables were encoded as ordered integers, and binary variables were coded as 0/1. Missing data were imputed using the median (continuous/ordinal) or mode (binary). Cluster solutions were tested for k = 2–8, and the five-cluster solution (k = 5) was selected for offering the best balance between statistical robustness and clinical interpretability, as empirically validated in the Section S1 (Section S1D).

### 2.6. Ethical Considerations

The study was conducted in strict adherence to the ethical principles for medical research involving human subjects as outlined in the Declaration of Helsinki. The study was approved by the Ethics Committee for Clinical Research of Hospital Clínico San Carlos (Ref. 23/549- E, approved in Madrid, 5 September 2023). Written informed consent was obtained from all participants prior to enrollment. Data collection and processing complied with the principles of the Declaration of Helsinki and applicable European and national data protection regulations. All patient identifiers were anonymized prior to any statistical processing.

## 3. Results

### 3.1. Cohort Characteristics and the Burden of Poor Clinical Control

The final cohort for analysis included 988 patients. Table 1 summarizes the overall cohort. The study population consisted of elderly patients, predominantly men, with a significant history of tobacco exposure. A high disease burden was observed, with moderate to severe airflow limitation and a high symptom burden. Treatment regimens were often complex, with frequent non-adherence.

**Figure 1:**
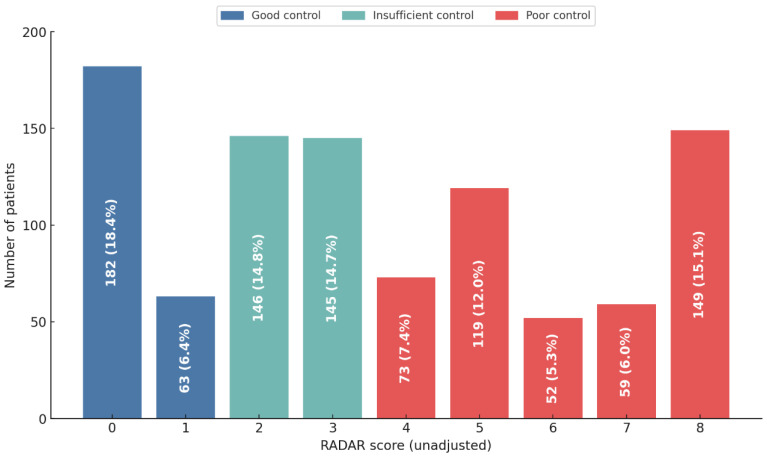
Distribution of Patients by RADAR Score. Note. The bar chart shows the number of patients (N = 988) at each score level for the RADAR score. The color of the bars indicates the clinical control category: blue for ‘Good control’, teal for ‘Insufficient control’, and red for ‘Poor control’. Abbreviations: FEV1, forced expiratory volume in 1 s; RADAR, Rescue medication, Acute exacerbations, Dyspnea, physical Activity, and Risk.

Patients were stratified into three groups based on their degree of clinical control as measured by the RADAR score. A minority of the cohort achieved ‘Good control’ (24.8%, *n* = 245), whereas a substantial proportion demonstrated ‘Insufficient control’ (29.5%, *n* = 291). Critically, the largest segment of the population, representing nearly half of the patients, was classified as having ‘Poor control’ (45.7%, *n* = 452). Figure 1 illustrates the patient distribution across each individual RADAR score from which these control categories are derived. Notably, this classification remained unchanged when using the FEV_1_-adjusted RADAR score (Supplementary Figure S2).

Table 2 presents a detailed comparative analysis of these characteristics stratified by clinical control status. A clear and statistically significant gradient of worsening clinical severity was observed as control declined from ‘Good’ to ‘Poor’ (Table 2). This trend was evident across multiple domains, including the distribution of clinical phenotypes; a detailed comparison of these is provided in Supplementary Figure S3. A detailed analysis of factors linked to frequent rescue inhaler use is available in Supplementary Figure S4. This pattern of clinical decline was mirrored by functional status, behavioral factors, and treatment regimens. Poorer control was significantly associated with a higher prevalence of active smoking (33.8% vs. 25.2%), greater comorbidity burden (Charlson index ≥ 2: 75.9% vs. 58.7%), therapeutic non-adherence, and a mixed pattern of non-adherence (30.3% vs. 15.4%). Furthermore, patients with poor control had a significantly higher daily dosing frequency (53.3% vs. 37.1%) and a greater inhaler burden. Notably, specific device regimens were also linked to control status; for instance, the use of a single spacer was significantly more prevalent in the poor control group (3.1% vs. 0.6%, *p* = 0.002).

**Figure 2:**
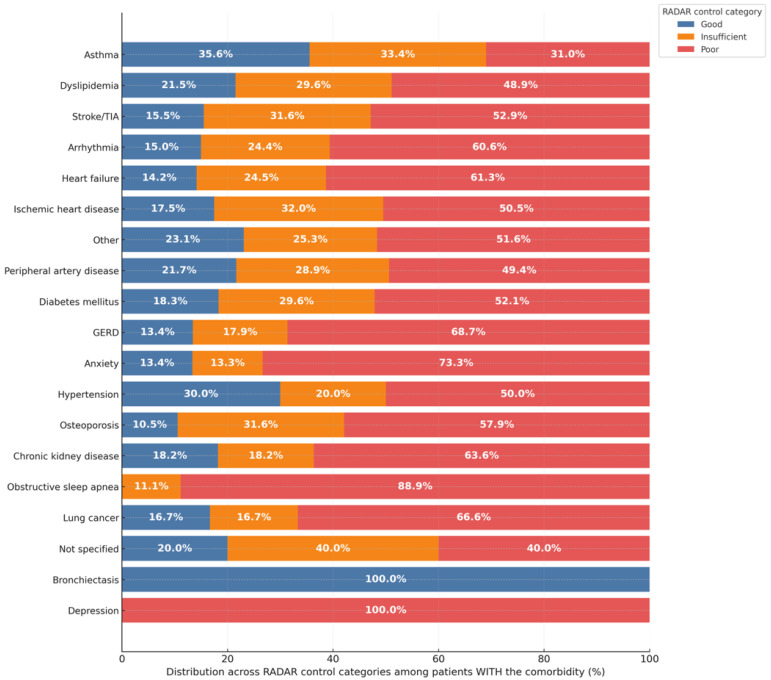
Distribution of Clinical Control Status by Comorbidity. Note. The stacked horizontal bar chart shows the proportional distribution of patients across the three RADAR control categories for each listed comorbidity. For each comorbidity, the bar is normalized to sum to 100%. Each segment displays the percentage of patients with that comorbidity who were classified as having ‘Good’ (blue), ‘Insufficient’ (orange), or ‘Poor’ (red) clinical control. The raw data, including the absolute number of patients (*n*) for each segment, are available in Supplementary Table S1.

As shown in Figure 2, the distribution of clinical control varied substantially across different comorbidities, revealing a clear pattern where certain conditions are associated with a much higher burden of poor control. This trend was most extreme among patients with depression, where all were classified as having ‘Poor control’, and in those with obstructive sleep apnea, where nearly 90% fell into this category. Similarly, a high prevalence of poor control was observed in patients with anxiety (73.3%), gastroesophageal reflux disease (68.7%), heart failure (61.3%), and arrhythmia (60.6%). The association between specific comorbidities such as anxiety, gastroesophageal reflux disease, heart failure, arrhythmia, and obstructive sleep apnea and a higher prevalence of poor control was statistically significant (all *p* ≤ 0.030). In contrast, other conditions demonstrated different patterns. Patients with asthma were more evenly distributed across the control categories, while notably, all patients with bronchiectasis were classified as having ‘Good control’.

A detailed analysis of the Test of Adherence to Inhalers (TAI) revealed a clear gradient of worsening adherence behaviors in patients with poorer clinical control (Supplementary Figure S5). Across nearly all items, the proportion of patients reporting optimal adherence (“Never” engaging in non-adherent behavior) decreased as control status declined from ‘Good’ to ‘Poor’.

This trend is highlighted by specific non-adherent behaviors. For instance, when asked if they forgot to use their inhaler (TAI2), the cumulative prevalence of non-adherent responses (‘Sometimes’, ‘Almost always’, or ‘Always’) more than doubled from 15.1% in the ‘Good control’ group to 34.4% in the ‘Poor control’ group (*p* < 0.001). A similar and significant pattern was observed for intentionally stopping medication when feeling better (TAI3), where the prevalence of non-adherence increased from 20.7% in the ‘Good control’ group to 30.3% in the ‘Poor control’ group (*p* < 0.001). The complete distribution of responses for all TAI items is provided in Supplementary Table S2.

### 3.2. Independent Determinants of Poor Clinical Control

The results of the multivariable logistic regression analysis, detailed in Table 3, identified several independent determinants of poor clinical control. Diagnostic metrics confirmed the model’s statistical validity (Section S1C, Tables S9 and S10), showing an Area Under the Curve (AUC) of 0.794, a non-significant Hosmer-Lemeshow test (*p* = 0.808). The calibration plot showed concordance between predicted and observed risks (Section S1C, Figure S7). Decision Curve Analysis (DCA) indicated a positive net benefit for the model across threshold probabilities ranging from approximately 10% to 80% (Section S1C, Figure S8). Variance Inflation Factors were <1.75 for all predictors. Markers of underlying disease severity were the strongest predictors. The eosinophilic exacerbator phenotype (aOR 6.85, *p* < 0.001) and the non-eosinophilic exacerbator phenotype (aOR 4.91, *p* < 0.001) were associated with the highest odds of poor control, followed by severe airflow limitation (FEV_1_ < 50%; aOR 2.61, *p* < 0.001) and a higher comorbidity burden (Charlson Index; aOR 1.26, *p* < 0.001).

Critically, the model also confirmed the role of several modifiable factors. Active smoking (aOR 1.92, *p* < 0.001), poorer therapeutic adherence (TAI-12 score; aOR 0.96, *p* = 0.014), a high dosing frequency (≥4 inhalations/day; aOR 1.54, *p* = 0.009) and a high inhaler burden (≥3 devices) (aOR 1.84, *p* = 0.004) were all independently associated with a higher likelihood of poor control. The prevalence of poor control stratified by these key factors is detailed in Supplementary Figure S6.

**Figure 3:**
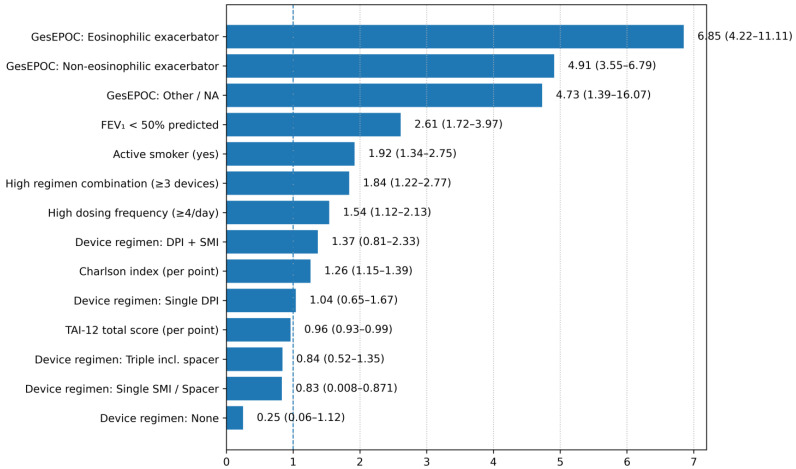
Independent Determinants of Poor Clinical Control. Note. The figure illustrates adjusted odds ratios (aORs) and 95% confidence intervals derived from the final multivariable logistic regression models evaluating poor clinical control (RADAR score ≥ 4). Predictors are ordered by effect magnitude. An aOR greater than 1 indicates increased odds of poor control, whereas values below 1 indicate a protective association. Abbreviations: DPI, dry powder inhaler; FEV_1_, forced expiratory volume in 1 s; GesEPOC, Spanish COPD Guidelines; pMDI, pressurized metered-dose inhaler; SMI, soft-mist inhaler.

Figure 3 displays the independent determinants of poor clinical control identified in the corrected multivariable model, ranked according to the magnitude of their association. Markers of underlying disease severity and exacerbation phenotype emerged as the strongest predictors of poor control. Specifically, the eosinophilic exacerbator phenotype showed the highest odds of poor clinical control (aOR 6.85), followed by the non-eosinophilic exacerbator phenotype (aOR 4.91).

Additional significant risk factors included severe airflow limitation (FEV_1_ < 50% predicted; aOR 2.61), active smoking (aOR 1.92), and increasing comorbidity burden as measured by the Charlson index (aOR 1.26 per point). Treatment-related behavioral factors also showed independent associations, including higher dosing frequency (≥4 inhalations/day; aOR 1.54) and lower treatment adherence (TAI-12 score per point; aOR 0.96). The use of multiple inhaler devices (≥3 devices) was associated with increased odds of poor control in the stepwise model (aOR 1.84).

### 3.3. The Clinician Perception Gap and In-Depth Analysis of Actionable Drivers

**Figure 4:**
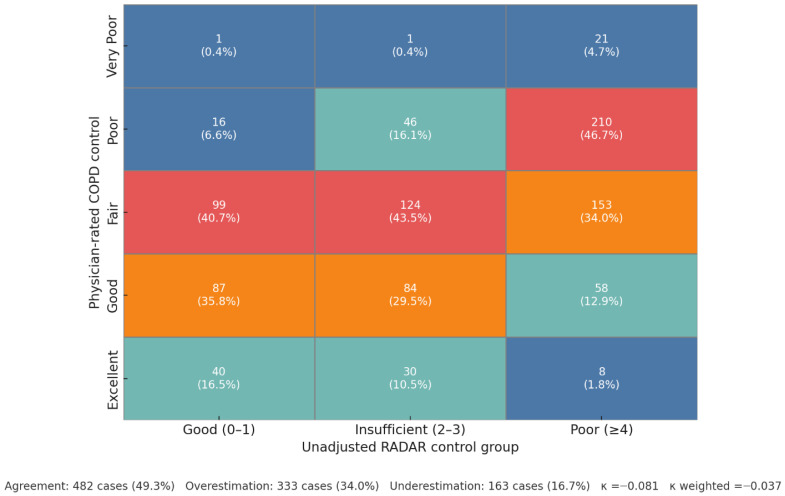
Concordance Between Physician-Assessed and RADAR-Classified Clinical Control (5 × 3 Classification). Note. Cross-tabulation of patient distribution (N = 978) according to the five-level physician-rated control category (*y*-axis) and the three-level unadjusted RADAR control group (*x*-axis). The data presented here are corrected for internal consistency; the absolute numbers (*n*) and column totals have been validated against the primary analysis cohort. Each cell displays the absolute number of patients and the corresponding column percentage. The footer displays the Cohen’s kappa (κ) and weighted kappa statistics, assessing the level of agreement between the two classification methods.

The analysis of the concordance between the physician’s subjective assessment and the objective RADAR classification revealed a profound perception gap (Figure 4). While direct agreement between the two methods was found in 49.3% of cases (*n* = 482), the remaining discordance was asymmetrical, with physicians overestimating control more frequently than underestimating it (34.0% vs. 16.7% of cases, respectively). This perception gap was most pronounced in the highest-risk population: among the 450 patients objectively classified with ‘Poor’ control, physicians still assigned a more favorable rating (Excellent, Good, or Fair) in nearly half of these cases (48.7%, *n* = 219). Consequently, the overall agreement was exceptionally poor, as quantified by the low statistical concordance (Cohen’s κ = −0.081; weighted κ = −0.037).

### 3.4. Identification of Novel Patient Phenotypes Within the Poorly Controlled Population

**Figure 5:**
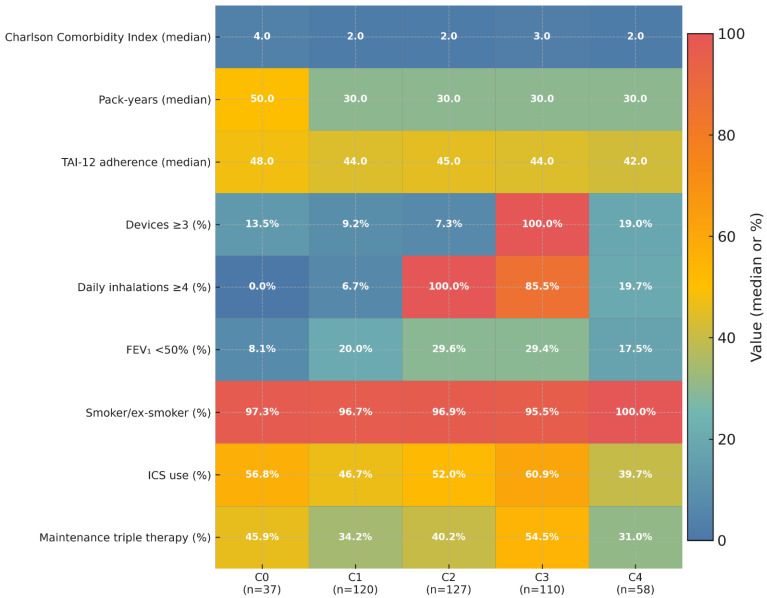
Heatmap of Patient Characteristics Across Five Clusters. Note. Heatmap of the five patient clusters (k-medoids; N = 452 with RADAR ≥ 4). Columns correspond to clusters (sample sizes shown), and rows represent clinical, behavioral, and treatment-related variables. Cell color reflects the value of each variable (median for continuous; percentage for categorical), with the color scale normalized to 0–100 for visualization. Annotated values are medians or percentages, respectively. Abbreviations: FEV_1_, forced expiratory volume in 1 s; ICS, inhaled corticosteroid; IQR, interquartile range; TAI, Test of Adherence to Inhalers. Source data: Supplementary Table S3.

To better understand the heterogeneity of the poorly controlled COPD cohort, a k-medoids cluster analysis was performed. The analysis identified five distinct and clinically meaningful patient phenotypes, whose defining features are visualized in Figure 5 and detailed in Supplementary Table S3.

The primary drivers separating these phenotypes were found to be treatment complexity and patient comorbidity burden, rather than traditional markers like the severity of airflow limitation. The analysis revealed two clusters primarily defined by patient history: a Multimorbid Exacerbator group (C0) with the highest burden of comorbidity and a Smoker-Dominant Frequent Exacerbator group (C1). In contrast, two other phenotypes were distinguished by their treatment patterns: a High Inhalation-Burden group (C2), characterized by a high number of daily puffs from few devices, and a Device-Intense Severe Patients group (C3) with maximum treatment complexity involving both multiple devices and frequent inhalations. Finally, a fifth phenotype was identified, the Lower-Risk, Poor Adherence group (C4), representing patients with lower comorbidity and symptom burden, but with the poorest adherence.

### 3.5. Hierarchical Importance of Predictors: A Machine Learning Analysis

**Figure 6:**
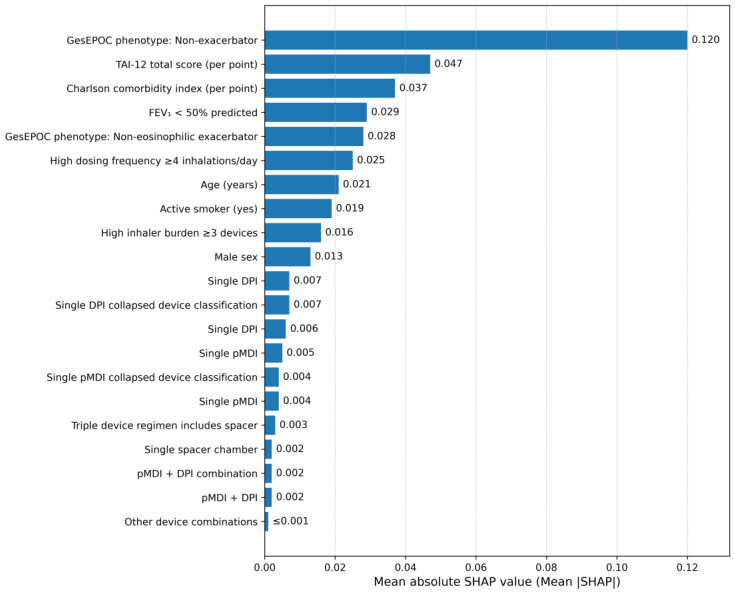
Hierarchical Importance of Predictors based on Mean Absolute SHAP Values. Note. The bar chart ranks the predictors based on their global importance, measured by the mean absolute SHAP value displayed on the *x*-axis. Longer bars indicate a greater overall contribution of the feature to the model’s prediction of poor clinical control.

To obtain a quantitative ranking of predictor influence using a non-linear approach, a Random Forest classifier was implemented. Figure 6 displays the hierarchy derived from the analysis of mean absolute SHAP (SHapley Additive exPlanations) values.

The GesEPOC clinical phenotype emerged as the dominant determinant of poor control, exhibiting the highest mean absolute SHAP value (0.120). This was followed by treatment adherence (TAI-12 total score), which ranked as the second most influential predictor (0.120 vs. 0.047). The Charlson Comorbidity Index (0.037) and severe airflow limitation (FEV_1_ < 50%; 0.029) occupied the third and fourth positions, respectively.

Consistent with the regression models, demographic factors (age, sex) and specific device configurations (e.g., single vs. multiple devices) showed minimal contribution to the model’s predictive power once clinical severity and adherence were accounted for. The complete numerical data for all predictors, including the validation via Permutation Importance, are listed in the Section S1 (Table S11 and Figure S9).

## 4. Discussion

In this large, real-world cohort of COPD patients managed in primary care with inhaled maintenance treatment using various devices, we demonstrate that suboptimal clinical control is highly prevalent and driven by a complex interplay of disease severity and, critically, several actionable, treatment-related factors. Our study provides relevant results that emphasize the importance of evaluating the clinical control of COPD and acting on modifiable factors in the treatment of COPD: first, we quantify a profound perception gap between clinicians’ subjective assessments and patients’ objective control status, a finding that likely fuels therapeutic inertia; second, we identify treatment complexity and poor adherence as key modifiable determinants of poor control; and third, we deconstruct the heterogeneity of this poorly controlled population (RADAR ≥ 4) by identifying five distinct and clinically actionable patient phenotypes.

### 4.1. The Clinician Perception Gap: A Major Barrier to Effective COPD Management

Perhaps the most striking finding of our study is the quantification of a profound clinician perception gap. We found that in 48.7% of cases objectively classified with ‘Poor Control’ by the validated RADAR score, the treating physician rated the patient’s control as ‘Good/Excellent’ or ‘Moderate’, with an overall agreement worse than chance (Figure 4). This finding provides a powerful, data-driven explanation for the high rates of therapeutic inertia widely reported in COPD management [17]. Indeed, data from the same EPOCONSUL audit have previously shown therapeutic inertia in 62.2% of uncontrolled patients undergoing follow-up in pulmonology clinics, an inaction strongly associated with the physician’s erroneous perception that the patient was, in fact, controlled [18]. While other studies, such as the MIRROR survey, have highlighted general discrepancies between patient and physician perceptions of symptom burden [22], our study’s key contribution is demonstrating this gap using a validated, objective control assessment tool as the benchmark. This suggests that without the systematic implementation of such tools, clinicians may be operating with a fundamentally flawed assessment of their patients’ status, a challenge consistent with prior evidence showing that reliance on subjective judgment has also contributed to disparities in COPD diagnosis [23,24,25]. Collectively, these findings underscore that a “treat-to-target” strategy will remain unattainable in routine practice unless objective, standardized tools are routinely incorporated into clinical workflows.

### 4.2. Deconstructing Poor Control: The Vicious Cycle of Treatment Complexity and Non-Adherence

Our regression model reinforces the central role of modifiable, treatment-related factors, supporting the “vicious cycle” hypothesis. The independent association of a high dosing frequency (≥4 inhalations/day) with poor control aligns with a substantial body of evidence (Table 3). This result is supported by evidence demonstrating that the complexity of the regimen (more inhalations and devices) increases errors and reduces clinical efficacy [26,27]. Inhaler polypharmacy is common in patients with multimorbidity [28] and has been consistently linked to both poorer adherence and a higher frequency of critical errors in inhalation technique [26]. That is why, when monitoring patients with COPD, if the response to treatment is inadequate and clinical control is not achieved, guidelines recommend reviewing adherence and inhalation technique before modifying therapy. In this process, it is recommended to consider simplifying the therapeutic regimen, using combinations of drugs in a single device to reduce complexity. Real-world studies show that open triple therapy, using two or three inhalers (MITT) still accounts for a significant proportion of triple therapy prescriptions in COPD, although the trend is toward the use of single devices. A retrospective observational study in Spain (BIG-PAC) showed that, between 2018 and 2019, most patients who started triple therapy did so with MITT, representing approximately 78% of cases compared to 22% who started single-inhaler triple therapy (SITT) [29]. This reflects that, during that period, open triple therapy was the predominant modality in Spanish primary care, mainly due to the limited availability of single devices and factors related to access and cost. Single-inhaler triple therapy is associated with improved treatment persistence, adherence, and reduced health care resource utilization in real-world practice, which translates to lower exacerbation rates and mortality compared to open-label triple therapy [30,31,32]. Therefore, simplifying treatment by reducing the number of inhalations and devices improves adherence and clinical control in COPD, which is particularly relevant in primary care in Spain, where the transition to single devices is underway. Crucially, and in contrast to univariate findings, our adjusted model identified that a high inhaler burden (≥3 devices) is a significant independent driver of poor control (aOR 1.84). Regarding device type, previous studies have consistently shown that patients using pMDIs with a spacer frequently make critical errors [33,34,35], often due to patient-related factors such as advanced age, cognitive impairment, or sensory deficits [36,37], coupled with insufficient training [38]. However, our multivariable analysis clarifies that once these factors and regimen complexity are accounted for, the specific device type is less determinative than the overall burden of the regimen. This suggests that the “difficulty” often attributed to specific devices like spacers may largely reflect the challenge of managing a complex, multi-device regimen. Additionally, the prescription of spacers likely acts as a surrogate marker for “difficult-to-treat” populations—such as the elderly or those with physical limitations—creating a “confounding by indication” that is mitigated when the overall regimen burden is rigorously adjusted for.

Furthermore, our study not only confirms poor therapeutic adherence as an independent determinant of poor control—a finding consistent with extensive literature showing that adherence is often suboptimal [26,27] and directly correlates with an increased risk of exacerbations and mortality [28]—but also characterizes the specific non-adherent behaviors that drive this association. The analysis of the TAI responses revealed a clear dose–response relationship: as clinical control worsened from ‘Good’ to ‘Poor’, the prevalence of optimal adherence (“Never” engaging in non-adherent behaviors) systematically decreased across nearly all domains (Supplementary Figure S5). This was particularly evident for key behaviors such as forgetting to use the inhaler (TAI2), where the cumulative prevalence of non-adherence more than doubled between the ‘Good’ and ‘Poor’ control groups. By providing this granular insight, our findings underscore the importance of moving beyond simply measuring adherence to identifying and addressing the specific behavioral patterns that undermine treatment efficacy.

### 4.3. Beyond a Monolithic View: Actionable Phenotypes for Personalized COPD Care

Personalized therapeutic strategies in COPD, particularly regarding inhaled triple therapy, are increasingly emphasized in clinical practice guidelines, which recommend individualizing inhaler regimens based on symptom burden, exacerbation risk, blood eosinophil count, comorbidities, inhaler technique, and patient preferences [3,4]. Large cohort analyses, such as COPDGene and SPIROMICS, have used advanced analytic methods to delineate clinically distinct COPD phenotypes, highlighting the dynamic nature of the disease and the need for ongoing reassessment [39]. Effective personalization involves selecting the simplest regimen and device that suit the patient’s abilities, ensuring proper training, and periodically reviewing adherence and inhalation technique to optimize control. Our data-driven approach moves beyond traditional classifications by using cluster analysis to identify clinically meaningful subgroups that may benefit from targeted strategies [40].

Our analysis identified five reproducible and clinically meaningful phenotypes within the poorly controlled cohort, as visualized in Figure 5. A key finding was that heterogeneity in this population is driven less by lung function or smoking status and more by treatment complexity, comorbidities, and adherence behavior. The five identified phenotypes represent distinct clinical challenges: C0 (Multimorbid Exacerbators) is defined by the highest comorbidity burden and heavy smoking exposure; C1 (Smoker-Dominant Frequent Exacerbators) exhibits nearly universal smoking and frequent exacerbations; C2 (High Inhalation Burden, Simple Devices) is characterized by a high number of daily puffs from few devices; C3 (Device-Intense Severe COPD) represents the opposite end of treatment complexity with universal use of multiple devices and high inhalation frequency; and C4 (Lower-Risk, Poor Adherence) combines a lower comorbidity and risk profile with the poorest adherence. These findings suggest that among patients already classified as poorly controlled, management patterns and multimorbidity are the dominant factors in defining patient subgroups.

### 4.4. Hierarchy of Influence: Insights from Machine Learning

While our logistic regression models identified significant associations, the application of Machine Learning (SHAP) enabled us to establish a precise, scale-independent hierarchy of influence. This analysis (Figure 6) revealed a dual driver of poor control: structural biological severity and patient behavior. Specifically, the GesEPOC clinical phenotype emerged as the dominant structural determinant. However, crucially, adherence behavior (TAI-12) ranked as the second most influential factor in the SHAP analysis, surpassing other traditional severity markers such as comorbidities or FEV_1_. This finding reinforces the concept that “treatment failure” is often behavioral rather than purely pharmacological or functional. Consequently, clinical strategies that prioritize escalating drug complexity without first addressing the “adherence barrier” are likely to yield limited returns, highlighting the necessity of integrating behavioral assessment into routine risk stratification, as recommended by current strategic updates [3,4].

### 4.5. Limitations and Methodological Considerations

We acknowledge several limitations. First, the cross-sectional approach to analyzing data from the inclusion visit and previous history data allows us to establish strong associations but does not permit causal inference. This is particularly relevant when interpreting associations with treatment regimens, where the possibility of confounding by indication cannot be excluded. Second, regarding missing data, while 4.9% of cases had missing values in some covariates, we utilized Multiple Imputation (MI) to handle this limitation. Our pooled estimates were highly consistent with complete-case analyses (Section S1: Table S8), confirming that missingness did not distort our core findings.

Third, our cohort was drawn from primary care system, and the results may be influenced by local clinical guidelines and healthcare structures. Therefore, the generalizability of the RADAR score and the identified phenotypes to other healthcare systems or specialized care settings requires further validation. Fourth, adherence was measured via the TAI, a self-report questionnaire, which may be subject to recall or social desirability bias. Nevertheless, the TAI is a well-validated and reliable instrument for this purpose [20]. Fifth, regarding the interpretation of the regression model, while adjusted odds ratios provide clinically actionable estimates of association strength, they should not be directly compared as a hierarchy of predictive importance, given the differing scales of the predictor variables. However, we specifically addressed this methodological limitation by implementing a Random Forest classifier with SHAP analysis, which allowed us to establish a robust and scale-independent hierarchy of predictor importance. Conversely, the study is strengthened by its large sample size (N = 988), its multicenter nature, and its real-world setting, which collectively enhance the external validity of our findings. The methodological rigor, including the use of a validated primary outcome measure (the RADAR score) and a robust, well-calibrated regression model, underpins the internal validity of our conclusions.

### 4.6. Clinical Implications and Future Directions

The findings of this study have significant and immediate implications for clinical practice. First and foremost, they issue a clear call to action against relying solely on subjective clinical impression to assess COPD control. The profound perception gap we have quantified underscores the urgent need for the routine integration of simple, objective, and validated tools like the RADAR score into clinical workflows to unmask the true burden of poor control and combat therapeutic inertia [12].

Second, our results advocate for a paradigm shift in the management of the poorly controlled patient. Before automatically escalating pharmacotherapy, clinicians should perform a systematic assessment of modifiable factors. This involves evaluating treatment complexity with a view to simplification where possible, formally assessing therapeutic adherence, and verifying correct inhalation technique. This “simplify and verify” approach represents a more targeted and evidence-based strategy than simply adding more medication, which our data suggest could be counterproductive if complexity is the root cause of the problem.

Third, these results underscore the need for tailored interventions that address polypharmacy and treatment intensity, particularly in clusters with excessive device or inhalation burden (C2, C3). For multimorbid patients (C0), comprehensive comorbidity management may be more relevant than intensifying inhaler regimens. Conversely, C4 patients demonstrate that poor control can exist even with preserved function, emphasizing the importance of addressing poor adherence and optimizing treatment, rather than symptom-driven escalation (Figure 5; Supplementary Table S3).

Future research should focus on prospectively validating these findings. It will be essential to validate these phenotypes in different populations, especially in primary care, to determine the broader applicability of this personalized approach in the treatment of COPD. Longitudinal studies and intervention trials based on our results—for example, comparing a phenotype-guided treatment strategy with standard care—are needed to provide further evidence to optimize clinical practice toward a personalized approach to COPD treatment.

## 5. Conclusions

In this real-world cohort of patients with COPD, poor clinical control is highly prevalent and frequently unrecognized by treating physicians, creating a significant perception gap that likely perpetuates therapeutic inertia. Our findings underscore the necessity of integrating objective, validated tools into routine practice to accurately identify patients in need of intervention. The determinants of this poor control are multifactorial but are primarily driven by the clinical phenotype and adherence behavior, suggesting that a “simplify and verify” approach should precede therapeutic escalation. Furthermore, the heterogeneity of the poorly controlled COPD population, as revealed by the identification of five reproducible and clinically meaningful phenotypes (C0–C4), highlights that clinical phenotype, comorbidities, and adherence behavior are the dominant axes of separation. This calls for a shift from a monolithic management strategy to a more personalized, phenotype-guided approach. Collectively, these results provide a clear roadmap for improving COPD management by moving beyond subjective assessment toward an objective, evidence-based, and personalized standard of care.

## Figures and Tables

**Table 1:** Baseline Sociodemographic and Clinical Characteristics of the Study Cohort.

Characteristic	Total Cohort (*n* = 988)
Demographics	
Age, mean (SD), years	70.94 (10.04)
Gender (male), *n* (%)	607 (61.4)
Smoking History	
Active smoker, *n* (%)	287 (29.0)
Pack-years, median (IQR)	31 (20–45)
Comorbidities and Clinical Status	
Charlson Comorbidity Index ≥2, *n* (%)	655 (66.3)
Purulent sputum, *n* (%)	160 (16.2)
Physical activity <30 min/day, *n* (%)	449 (45.4)
Lung Function and Symptoms	
FEV_1_% predicted, *n* (%) *	
<50%	150 (15.3)
≥50% and <80%	667 (67.8)
≥80%	166 (16.9)
mMRC dyspnea score ≥2, *n* (%)	513 (51.9)
Rescue inhaler use ≥3 times/week, *n* (%)	394 (39.9)
Exacerbation History and Phenotype	
Exacerbations in prior year, median (IQR)	1 (0–2)
GesEPOC Phenotype, *n* (%) *	
Non-exacerbator	472 (48.5)
Exacerbator, non-eosinophilic	379 (38.9)
Exacerbator, eosinophilic	123 (12.6)
Treatment Regimen	
Number of devices, *n* (%) *	
2	706 (72.0)
≥3	274 (28.0)
Total inhalations ≥4 puffs/day, *n* (%)	434 (43.9)
Adherence and Technique	
TAI Adherence, *n* (%) *	
Poor	387 (46.3)
Intermediate	184 (22.0)
Good	265 (31.7)
Critical errors in technique, *n* (%)	179 (18.1)

Note. Data are presented as mean (SD), median (IQR), or *n* (%). Unless otherwise specified, percentages are calculated based on the total cohort (N = 988). SD: Standard Deviation; IQR: Interquartile Range; FEV_1_: Forced Expiratory Volume in 1 s; mMRC: modified Medical Research Council; GesEPOC: Spanish COPD Guidelines; TAI: Test of Adherence to Inhalers. * Percentages for these variables are calculated based on the number of patients with available.

**Table 2:** Patient Characteristics Stratified by RADAR Clinical Control Category (N = 988).

Domain/Characteristic	RADAR < 4 (*n* = 536)	RADAR ≥ 4 (*n* = 452)	*p*-Value
**Sociodemographic**			
Active smoker, *n* (%)	134 (25.2)	153 (33.8)	0.003
**Clinical Severity and Symptoms**			
FEV_1_ < 50% predicted, *n* (%)	44 (8.2)	106 (23.7)	<0.001
GesEPOC Phenotype, *n* (%)			<0.001
– Non-exacerbator	362 (68.3)	110 (24.8)	
– Exacerbator, non-eosinophilic	134 (25.3)	245 (55.2)	
– Exacerbator, eosinophilic	34 (6.4)	89 (20.0)	
High-risk GesEPOC phenotype, *n* (%)			<0.001
– Exacerbator (any)	168 (31.7)	334 (75.2)	
**Comorbidity and Functional Status**			
Charlson index ≥2, *n* (%)	314 (58.7)	341 (75.9)	<0.001
**Treatment-Related Factors**			
High dosing frequency (≥4 inh/day), *n* (%)	195 (37.1)	239 (53.3)	<0.001
High regimen burden (≥3 devices), *n* (%)	128 (24.1)	146 (32.6)	0.003
TAI adherence category, *n* (%)			<0.001
– Good (TAI ≥ 50)	172 (38.5)	93 (23.9)	
– Intermediate (46–49)	107 (23.9)	77 (19.8)	
– Poor (≤45)	168 (37.6)	219 (56.3)	
Non-adherence pattern (baseline), *n* (%)			<0.001
– Erratic	188 (42.1)	162 (41.6)	
– Mixed	69 (15.4)	118 (30.3)	
Behavioral–Structural phenotype (4 groups), *n* (%)			<0.001
– LowC + Good	171 (38.9)	197 (51.0)	
– HighC + Good	99 (22.5)	97 (25.1)	
– LowC + Poor	113 (25.7)	65 (16.8)	
– HighC + Poor	57 (13.0)	27 (7.0)	
High complexity (Core_TBI > 2), *n* (%)	181 (34.4)	141 (31.5)	0.331
**Device Type—Single Devices**			
– Single DPI	248 (46.3)	204 (45.1)	0.72
– Single pMDI	70 (13.1)	71 (15.7)	0.21
– Single SMI	9 (1.7)	4 (0.9)	0.28
– Single Spacer	3 (0.6)	14 (3.1)	0.002
**Multiple Device Types**			
– DPI + pMDI	92 (17.2)	77 (17.0)	0.94
– DPI + SMI	57 (10.6)	19 (4.2)	0.01
– pMDI + SMI	20 (3.7)	23 (5.1)	0.21
– pMDI + Spacer	6 (1.1)	7 (1.5)	0.64
– ≥3 device types	128 (24.1)	146 (32.6)	0.003

Note. Data are presented as median [interquartile range] or *n* (%). *p*-values are for comparisons across RADAR control groups unless otherwise indicated. For device regimen, *p*-values represent row-wise chi-square tests comparing each regimen vs. all others. Overall chi-square (all regimens): χ^2^(≈12) = 39.6, *p* < 0.001. Missing data were handled using Multiple Imputation by Chained Equations (MICE). Abbreviations: DPI, dry powder inhaler; FEV_1_, forced expiratory volume in 1 s; GesEPOC, Spanish COPD Guidelines phenotype classification; IQR, interquartile range; mMRC, modified Medical Research Council; pMDI, pressurized metered-dose inhaler; SMI, soft-mist inhaler; Spacer: Pressurized cartridge with chamber; TAI, Test of Adherence to Inhalers.

**Table 3:** Independent Determinants of Poor Clinical Control (RADAR ≥ 4). Multivariable Logistic Regression and LASSO Sensitivity Analysis.

Predictor	Univariate OR (95% CI)	Stepwise + Regimen aOR (95% CI)	LASSO-Refit aOR (95% CI)
Age (years)	1.01 (0.99–1.03), *p* = 0.351	—	—
Female sex	1.19 (0.89–1.58), *p* = 0.238	—	—
Active smoker (Yes)	1.78 (1.33–2.39), *p* < 0.001	1.92 (1.34–2.75), *p* < 0.001	1.93 (1.35–2.77), *p* < 0.001
Charlson index (per point)	1.27 (1.18–1.36), *p* < 0.001	1.26 (1.15–1.39), *p* < 0.001	1.27 (1.16–1.39), *p* < 0.001
GesEPOC phenotype (overall)	χ^2^ = 64.42, df = 2, *p* < 0.001	χ^2^ = 64.42, df = 2, *p* < 0.001	—
–Non-eosinophilic exacerbator	3.53 (2.67–4.67), *p* < 0.001	4.91 (3.55–6.79), *p* < 0.001	4.88 (3.53–6.75), *p* < 0.001
–Eosinophilic exacerbator	3.90 (2.52–6.04), *p* < 0.001	6.85 (4.22–11.11), *p* < 0.001	6.69 (4.22–10.61), *p* < 0.001
–GesEPOC: Other/NA	1.08 (0.35–3.34), *p* = 0.899	4.73 (1.39–16.07), *p* = 0.013	4.79 (1.42–16.22), *p* = 0.012
TAI-12 total score (per point)	0.94 (0.92–0.97), *p* < 0.001	0.96 (0.93–0.99), *p* = 0.014	0.96 (0.93–0.99), *p* = 0.011
FEV_1_ < 50% (Yes)	3.37 (2.32–4.90), *p* < 0.001	2.61 (1.72–3.97), *p* < 0.001	2.57 (1.70–3.89), *p* < 0.001
High dosing frequency (≥4/day)	2.06 (1.58–2.69), *p* < 0.001	1.54 (1.12–2.13), *p* = 0.009	1.54 (1.12–2.12), *p* = 0.008
High regimen combination (≥3 devices)	1.58 (1.14–2.19), *p* = 0.006	1.84 (1.22–2.77), *p* = 0.004	1.30 (0.92–1.84), *p* = 0.135
Device Regimen (ref: SINGLE pMDI)	χ^2^ = 18.84, df = 8, *p* = 0.016	χ^2^ = 18.84, df = 8, *p* = 0.016	—
–Triple/includes spacer	0.95 (0.64–1.41), *p* = 0.810	0.84 (0.52–1.35), *p* = 0.469	—
–None	0.21 (0.05–0.86), *p* = 0.030	0.25 (0.06–1.12), *p* = 0.070	—
–Single DPI	1.15 (0.78–1.71), *p* = 0.476	1.04 (0.65–1.67), *p* = 0.865	—
–Single SMI/Spacer	0.55 (0.15–2.04), *p* = 0.370	0.83 (0.008–0.871), *p* = 0.038	—
–DPI + SMI	1.60 (1.02–2.53), *p* = 0.041	1.37 (0.81–2.33), *p* = 0.005	—

Note. Logistic regression analysis predicting poor clinical control (RADAR score ≥ 4). Estimates are pooled from m = 20 imputed datasets (MICE). The multivariable model includes all predictors listed simultaneously. LASSO-refit aOR indicates estimates from a standard logistic regression refitted using the subset of predictors selected by cross-validated LASSO. Reference categories for key variables were as follows: High dosing frequency (ref = <4 inhalations/day); High inhaler burden (ref = <3 devices); Device Regimen (ref = Single pMDI); GesEPOC Phenotype (ref = Non-exacerbator). Abbreviations: aOR, Adjusted Odds Ratio; CI, Confidence Interval; FEV_1_, Forced Expiratory Volume in 1 s; GesEPOC, Spanish COPD Guidelines; OR, Odds Ratio; TAI, Test of Adherence to Inhalers.

## Data Availability

The datasets used and/or analyzed during the current study are available from the corresponding author on reasonable request.

## Supplementary Materials

The following supporting information can be downloaded at: https://www.mdpi.com/article/10.3390/jcm15031283/s1, Figure S1: Flow Diagram of Patient Selection for the Study; Figure S2: Distribution of Patients by FEV_1_-Adjusted RADAR Score; Figure S3: Distribution of Exacerbator and Non-Exacerbator Phenotypes Across Patient Characteristics; Figure S4: Distribution of Patient Characteristics According to Frequency of Rescue Inhaler Use; Figure S5: Patient-Reported Adherence Behaviors by Clinical Control Status; Figure S6: Prevalence of Poor Clinical Control by Modifiable Factors and Clinical Severity Indicators; Table S1: Cross-tabulation of Patient Comorbidities by RADAR Clinical Control Group (N = 988); Table S2: Distribution of TAI Adherence Responses by RADAR Control Group (N = 958–975); Table S3: Detailed Characteristics of the Five Patient Phenotypes (N = 452). Section S1: Methodological and Statistical Validation. Section S2: List of SIMPLIFY Study Investigators.

## Abbreviations

The following abbreviations are used in this manuscript:

COPD	Chronic Obstructive Pulmonary Disease
GesEPOC	Spanish COPD Guidelines
FEV_1_	Forced Expiratory Volume in 1 s
mMRC	Medical Research Council
IQR	Interquartile range
TAI	Test of Adherence to Inhalers
DPI	Dry powder inhaler
pMDI	Pressurized metered-dose inhaler
SMI	Soft-mist inhaler
ICS	Inhaled corticosteroid
