# Supplementary material for: Determinants and Phenotypes of Poorly Controlled COPD Using the RADAR Score: A Cohort in Real-World Primary Care
Source: J Clin Med. 2026 Feb 5;15(3):1283. doi: 10.3390/jcm15031283 (PMC12898748; doi:10.3390/jcm15031283)
Supplement: Supplementary file 1 [file jcm-15-01283-s001.zip › Supplementary Material.pdf]

Supplementary Material

Supplementary Figure S1. Flow Diagram of Patient Selection for the Study.

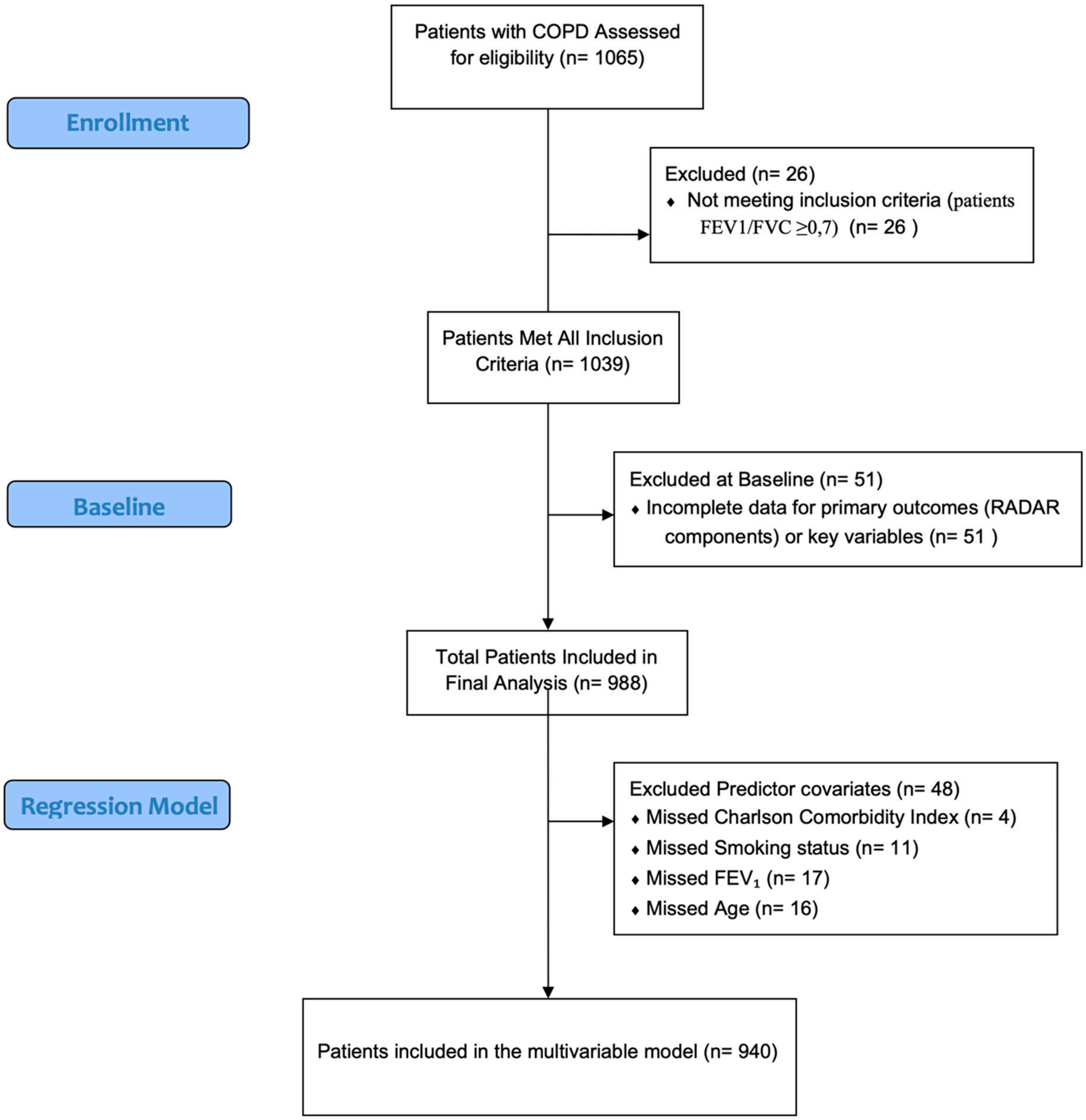

Note. The diagram details the flow of patients from the initial screening for eligibility to their inclusion in the final analytical cohort.

Supplementary Figure S2. Distribution of Patients by FEV_1-Adjusted RADAR Score

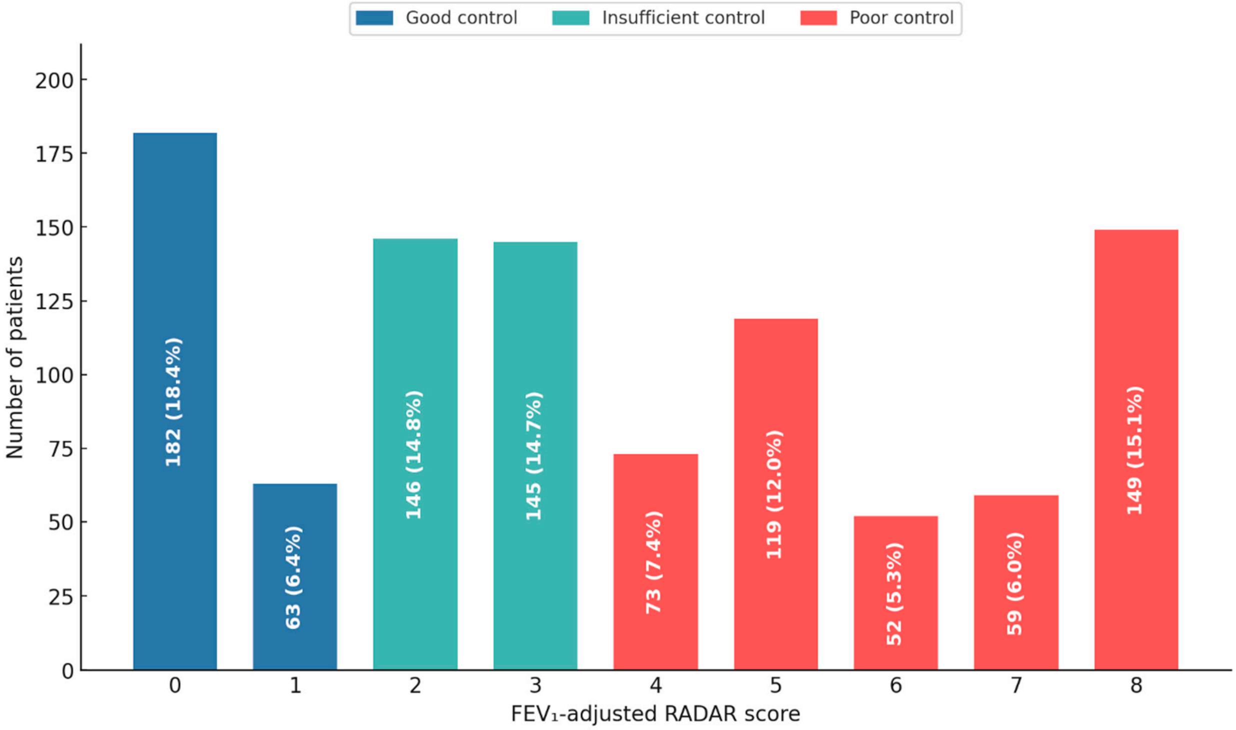

Note. The bar chart shows the number of patients (N = 988) at each score level for the FEV_1-adjusted RADAR score. The color of the bars indicates the clinical control category: blue for 'Good control', teal for 'Insufficient control', and red for 'Poor control'. Abbreviations: FEV_1, forced expiratory volume in 1 second; RADAR, Rescue medication, Acute exacerbations, Dyspnea, physical Activity, and Risk.

Bar chart showing the distribution of the patient cohort across each level of the FEV_1-adjusted RADAR score.

Supplementary Figure S3. Distribution of Exacerbator and Non-Exacerbator Phenotypes Across Patient Characteristics

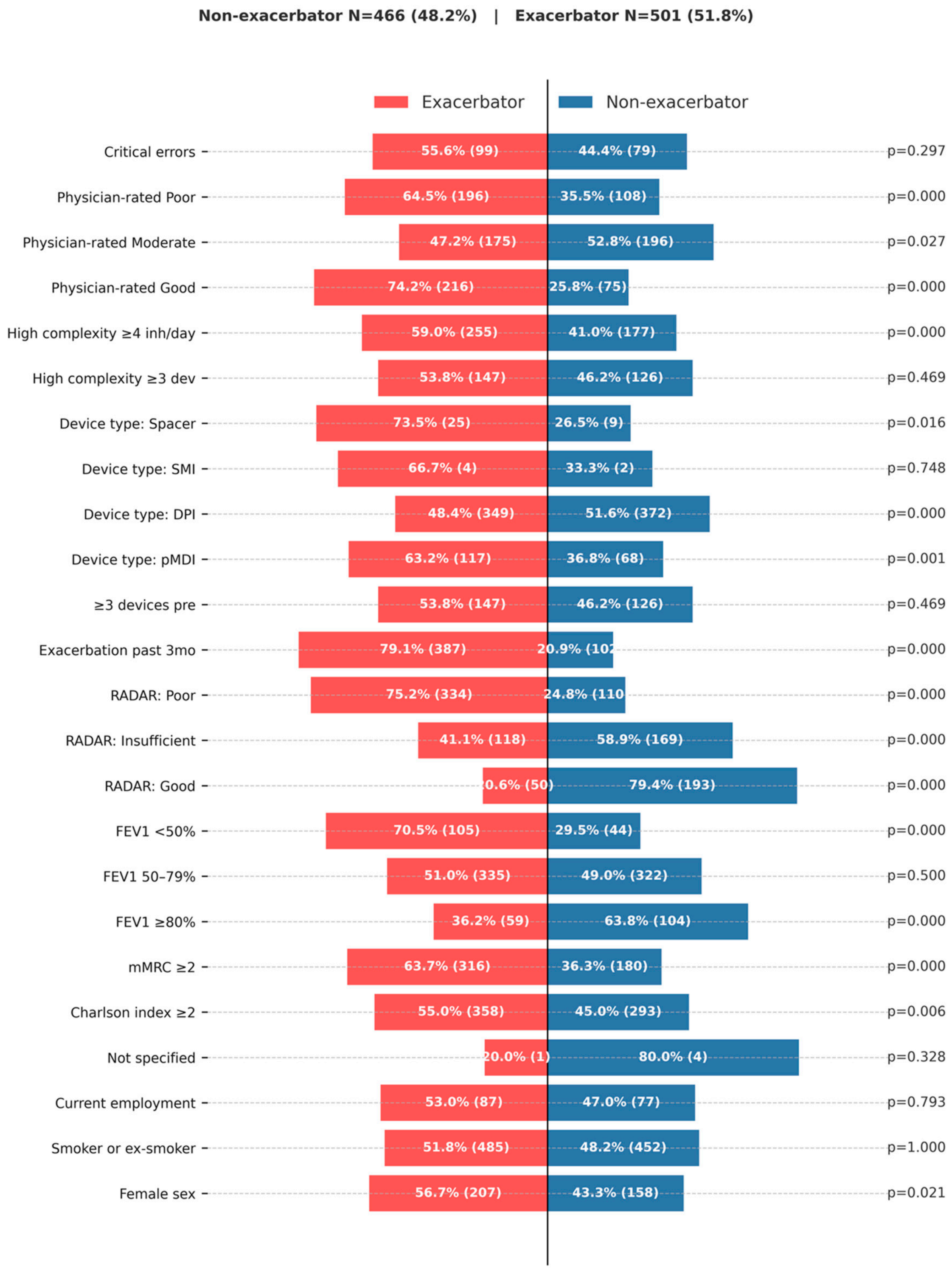

Note. The diverging bar chart shows the distribution of patient phenotypes for various characteristics in the subset of patients (N=967) with available phenotype data (excluding 1 patient classified as Other/NA). For each characteristic

listed on the y-axis, the bars represent the percentage of patients with that characteristic who were classified as 'Non-exacerbator' (blue) or 'Exacerbator' (red). Percentages sum to 100% per characteristic. p-values from Chi-square tests are shown on the right. Abbreviations: FEV_1, forced expiratory volume in 1 second; mMRC, modified Medical Research Council; pMDI, pressurized metered-dose inhaler; RADAR, Rescue medication, Acute exacerbations, Dyspnea, physical Activity, and Risk; SMI, soft mist inhaler.

As shown in Supplementary Figure **S**3, the distribution of exacerbator and non-exacerbator phenotypes differed significantly across numerous characteristics. The strongest associations were found with measures of disease stability and severity. For instance, patients with 'Poor' RADAR control were predominantly exacerbators, whereas those with 'Good' control were overwhelmingly non-exacerbators ($p < 0.001$).

Symptom burden and lung function also correlated with phenotype. Patients with significant dyspnea (mMRC ≥2) or severe airflow limitation (FEV_1 <50%) were far more likely to be exacerbators ($p < 0.001$ for both). Conversely, those with the most preserved lung function (FEV_1 ≥80%) were more likely to be non-exacerbators.

In contrast, no significant difference in phenotype distribution was found for several treatment-related or behavioral factors, including the presence of critical inhalation errors ($p = 0.297$), high treatment complexity ($p = 0.469$), or current smoking status ($p = 0.793$).

Supplementary Figure S4. Distribution of Patient Characteristics According to Frequency of Rescue Inhaler Use

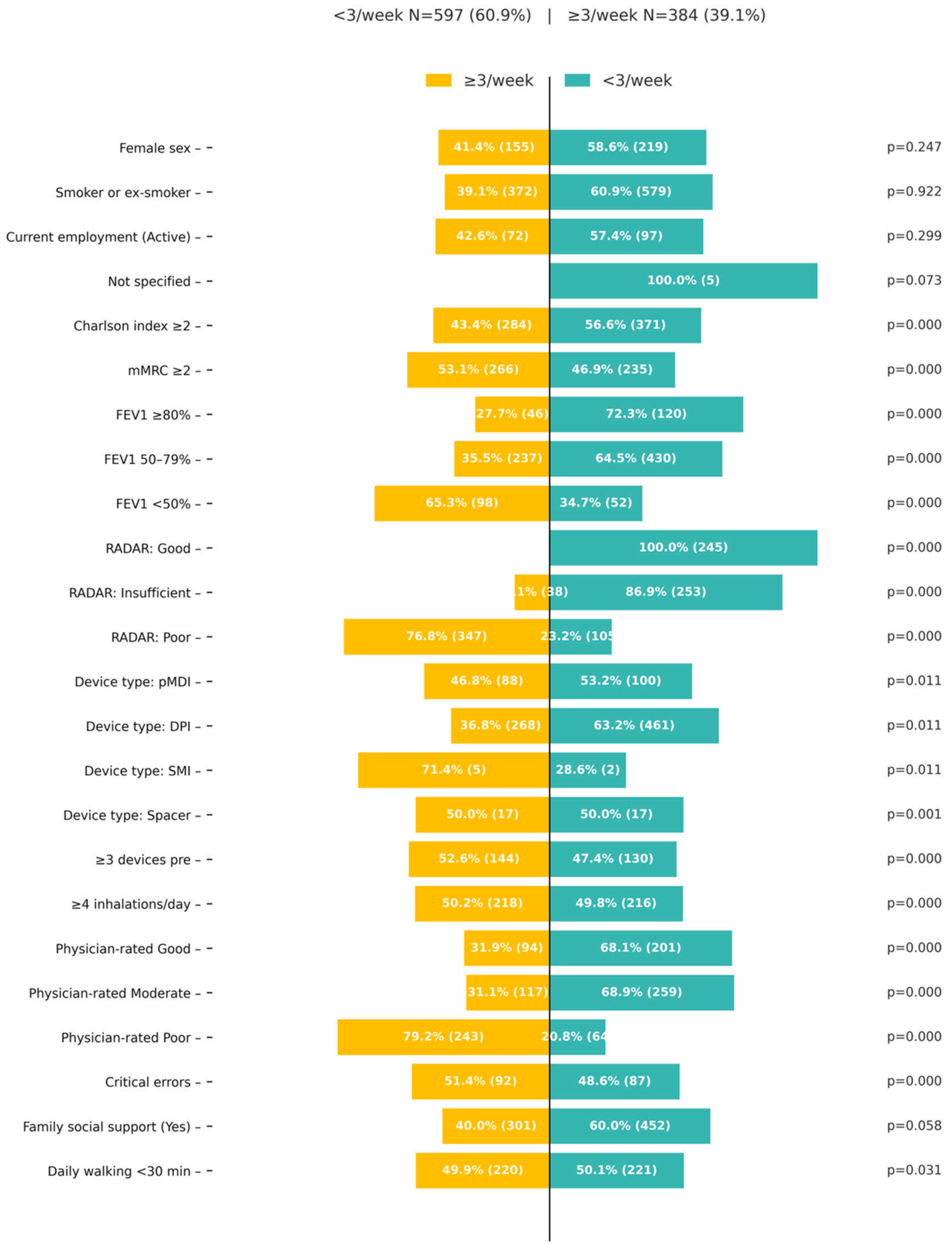

Note. The diverging bar chart compares the characteristics of patients who use their rescue inhaler frequently (≥3 times/week) versus infrequently (<3 times/week). The analysis is based on available data (N=981): frequent users N=384 and infrequent users N=597. For each characteristic on the y-axis, bars show the percentage of patients in each group. p-values from Chi-square tests are shown on the right. Abbreviations: FEV_1, forced expiratory volume in 1

second; mMRC, modified Medical Research Council; pMDI, pressurized metered-dose inhaler; RADAR, Rescue medication, Acute exacerbations, Dyspnea, physical Activity, and Risk; SMI, soft mist inhaler.

The frequency of rescue inhaler use was strongly associated with symptom burden, and disease severity (Supplementary Figure 4). 90.1% of patients in the frequent use group (≥3/week) had "poor" control, compared with only 17.4% in the infrequent use group (<3/week) ($p < 0.001$). This pattern was reflected in a significantly higher prevalence of dyspnea (mMRC ≥ 2), severe airflow limitation ($FEV_1 < 50\%$), and complex treatment regimens among frequent rescue inhaler users (all $p < 0.001$). In contrast, the frequency of rescue use was not significantly associated with several demographic or behavioral factors, including sex ($p = 0.247$), smoking history ($p = 0.922$), or family social support ($p = 0.518$).

Supplementary Table S1. Cross-tabulation of Patient Comorbidities by RADAR Clinical Control Group (N=988)

Comorbidity	Good (N=245)	Insufficient (N=291)	Poor (N=452)	χ^2 (df)	p-value	Cramer's V
Asthma	101 (41.2%)	95 (32.6%)	88 (19.5%)	39.78 (2)	<0.001	0.199
Ischemic heart disease	18 (7.3%)	33 (11.3%)	52 (11.5%)	3.31 (2)	0.191	0.051
Heart failure	15 (6.1%)	26 (8.9%)	65 (14.4%)	12.70 (2)	0.002	0.112
Arrhythmia	19 (7.8%)	31 (10.7%)	77 (17.0%)	14.00 (2)	0.001	0.117
Stroke/TIA	27 (11.0%)	55 (18.9%)	92 (20.4%)	10.01 (2)	0.007	0.093
Hypertension	6 (2.4%)	4 (1.4%)	10 (2.2%)	0.92 (2)	0.630	0.002
Dyslipidemia	48 (19.6%)	66 (22.7%)	109 (24.1%)	1.86 (2)	0.394	0.043
Diabetes mellitus	13 (5.3%)	21 (7.2%)	37 (8.2%)	1.98 (2)	0.372	0.044
Peripheral artery disease	18 (7.3%)	24 (8.2%)	41 (9.1%)	0.63 (2)	0.731	0.025
GERD	9 (3.7%)	12 (4.1%)	46 (10.2%)	15.24 (2)	<0.001	0.114
Osteoporosis	2 (0.8%)	6 (2.1%)	11 (2.4%)	2.25 (2)	0.325	0.045
Anxiety	6 (2.4%)	6 (2.1%)	33 (7.3%)	14.50 (2)	0.001	0.105
Depression	0 (0.0%)	0 (0.0%)	2 (0.4%)	2.38 (2)	0.305	0.044
OSA	0 (0.0%)	1 (0.3%)	8 (1.8%)	6.99 (2)	0.030	0.080
CKD	2 (0.8%)	2 (0.7%)	7 (1.5%)	1.45 (2)	0.483	0.032
Lung cancer	1 (0.4%)	1 (0.3%)	4 (0.9%)	1.07 (2)	0.585	0.028
Bronchiectasis	3 (1.2%)	0 (0.0%)	0 (0.0%)	9.13 (2)	0.010	0.082
Other	21 (8.6%)	23 (7.9%)	47 (10.4%)	1.48 (2)	0.478	0.030
Not specified	1 (0.4%)	2 (0.7%)	2 (0.4%)	0.27 (2)	0.873	0.001

Note. Data are presented as n (column %). The χ^2 value is from the Pearson Chi-square test, with degrees of freedom (df) in parentheses. Cramer's V is reported as a measure of effect size. Abbreviations: TIA, transient ischemic attack; GERD, gastroesophageal reflux disease; OSA, obstructive sleep apnea; CKD, chronic kidney disease.

Supplementary Table S1 presents the detailed cross-tabulation of patient comorbidities by the three RADAR clinical control categories ('Good', 'Insufficient', and 'Poor'). This table provides the absolute number of patients (n) and the corresponding column percentages that serve as the source data for the analysis presented in Figure 2 of the main manuscript.

Supplementary Figure S5. Patient-Reported Adherence Behaviors by Clinical Control Status

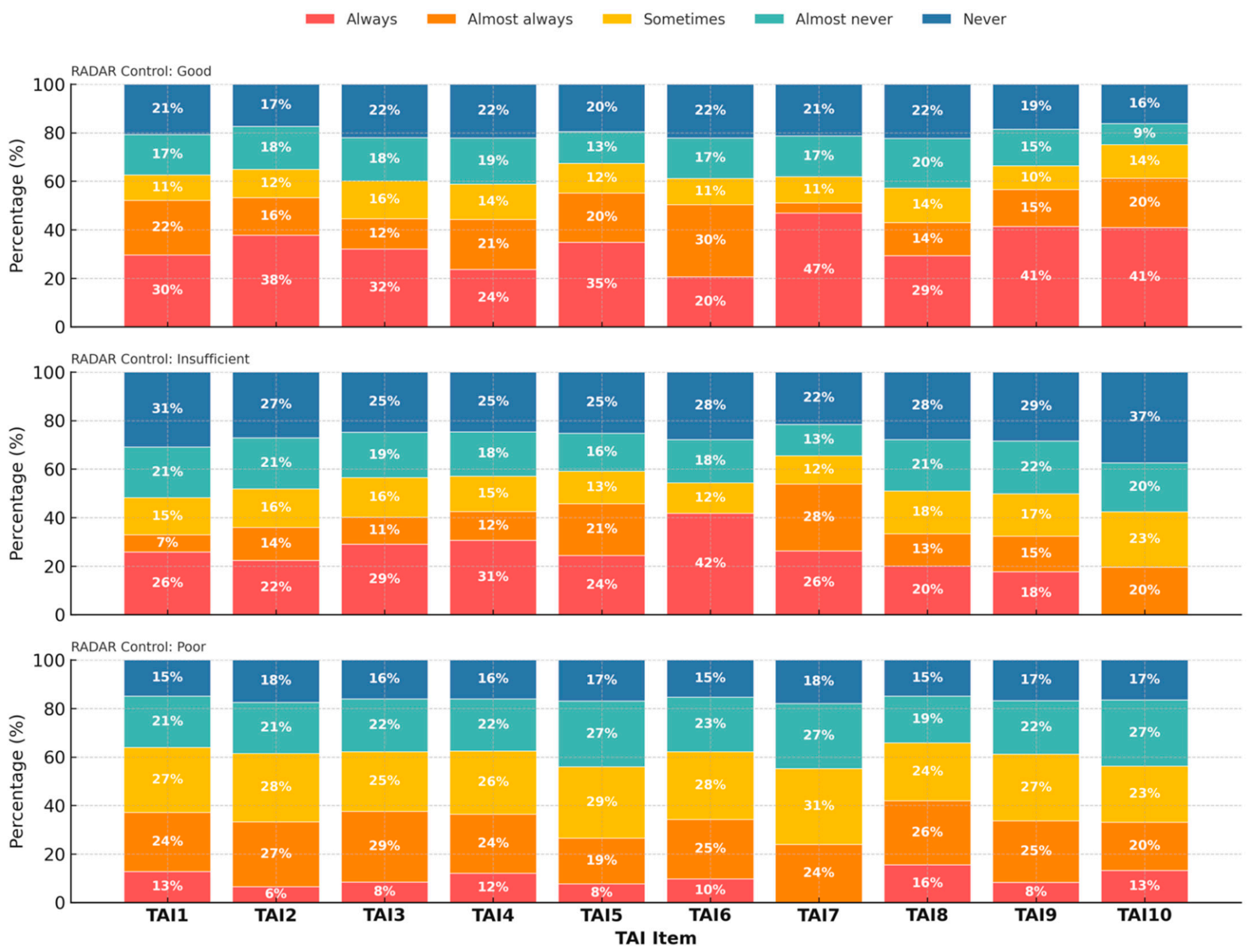

Note. The stacked bar charts show the distribution of patient responses for 10 of the 12 items from the Test of Adherence to Inhalers (TAI-12). Items 11 and 12 are omitted from this visualization due to their dichotomous (yes/no) response format. The results are stratified by RADAR clinical control group: 'Good' (top panel), 'Insufficient' (middle panel), and 'Poor' (bottom panel). The raw data are available in Supplementary Table S2.

This figure provides a detailed visualization of patient-reported adherence behaviors as measured by the Test of Adherence to Inhalers (TAI-12). The results are stratified into three panels according to the patient's clinical control status: 'Good' (top), 'Insufficient' (middle), and 'Poor' (bottom). Each stacked bar chart represents one TAI item and shows the proportional distribution of the five

possible patient responses, allowing for a direct visual comparison of adherence patterns across the different levels of COPD control. The raw numerical data for this figure are available in Supplementary Table S2.

Table Supplementary S2. Distribution of TAI Adherence Responses by RADAR Control Group (N = 958-975)

TAI Item & Response	Good	Insufficient	Poor	χ²(df)	p	Cramer's V
**Forgot inhaler when away from home**				46.07 (8)	<0.001	0.093
• Always	8 (3.3%)	6 (2.1%)	7 (1.6%)			
• Almost always	11 (4.5%)	3 (1.1%)	24 (5.4%)			
• Sometimes	8 (3.3%)	10 (3.5%)	41 (9.2%)			
• Almost never	55 (22.6%)	60 (21.1%)	142 (31.8%)			
• Never	161 (66.3%)	206 (72.3%)	233 (52.1%)			
**Forgot to use inhaler**				37.29 (8)	<0.001	0.107
• Always	4 (1.6%)	2 (0.7%)	24 (5.4%)			
• Almost always	2 (0.7%)	6 (2.1%)	20 (4.5%)			
• Sometimes	31 (12.8%)	36 (12.7%)	110 (24.5%)			
• Almost never	83 (34.2%)	84 (29.7%)	143 (32.1%)			
• Never	117 (48.1%)	155 (54.8%)	172 (38.6%)			
**Stopped taking medication when feeling well**				41.26 (8)	<0.001	0.109
• Always	8 (3.4%)	8 (2.8%)	4 (0.9%)			
• Almost always	7 (3.0%)	7 (2.5%)	31 (6.9%)			
• Sometimes	34 (14.3%)	39 (13.7%)	101 (22.5%)			
• Almost never	56 (23.6%)	65 (22.9%)	130 (29.0%)			
• Never	132 (55.7%)	165 (58.1%)	182 (40.6%)			
**Interrupted medication during holidays**				37.33 (8)	<0.001	0.121
• Always	4 (1.7%)	6 (2.1%)	4 (0.9%)			
• Almost always	9 (3.8%)	6 (2.1%)	21 (4.7%)			
• Sometimes	29 (12.3%)	34 (12.0%)	103 (23.1%)			
• Almost never	53 (22.6%)	59 (20.8%)	118 (26.5%)			
• Never	140 (59.6%)	179 (63.0%)	199 (44.7%)			
**Skipped medication when feeling down**				52.36 (8)	<0.001	0.127
• Always	3 (1.3%)	2 (0.7%)	1 (0.2%)			
• Almost always	7 (2.9%)	7 (2.5%)	10 (2.3%)			
• Sometimes	19 (7.9%)	20 (7.0%)	70 (15.9%)			
• Almost never	43 (17.9%)	49 (17.3%)	135 (30.8%)			
• Never	168 (70.0%)	206 (72.5%)	223 (50.8%)			
**Stopped medication due to side effects**				46.46 (8)	<0.001	0.147
• Always	1 (0.4%)	2 (0.7%)	1 (0.2%)			
• Almost always	4 (1.7%)	0 (0.0%)	7 (1.6%)			
• Sometimes	8 (3.3%)	9 (3.2%)	44 (10.0%)			
• Almost never	42 (17.5%)	44 (15.6%)	129 (29.1%)			
• Never	185	227	270			
**Lack of help with medication**				53.24 (8)	<0.001	0.149
• Always	3 (1.3%)	2 (0.7%)	0 (0.0%)			
• Almost always	1 (0.4%)	8 (2.8%)	10 (2.3%)			
• Sometimes	14 (5.9%)	18 (6.4%)	70 (15.8%)			
• Almost never	37 (15.6%)	34 (12.1%)	101 (22.7%)			
• Never	182 (76.8%)	219 (77.9%)	263 (59.2%)			
**Took fewer inhalations than prescribed**				38.34 (8)	<0.001	0.122
• Always	10 (4.2%)	7 (2.5%)	11 (2.5%)			
• Almost always	9 (3.8%)	9 (3.2%)	36 (8.1%)			
• Sometimes	27 (11.3%)	34 (12.1%)	94 (21.3%)			
• Almost never	61 (25.6%)	65 (23.1%)	119 (26.9%)			
• Never	131 (55.0%)	166 (59.1%)	182 (41.2%)			
**Medication interferes with daily life**				32.69 (8)	<0.001	0.122
• Always	3 (1.3%)	1 (0.4%)	1 (0.2%)			
• Almost always	4 (1.7%)	3 (1.1%)	11 (2.5%)			
• Sometimes	13 (5.5%)	18 (6.5%)	61 (13.7%)			
• Almost never	49 (20.7%)	55 (19.9%)	119 (26.8%)			
• Never	168 (70.9%)	200 (72.2%)	252 (56.8%)			
**Stopped inhaler due to financial constraints**				39.62 (8)	<0.001	0.105

• Always	2 (0.8%)	0 (0.0%)	1 (0.2%)			
• Almost always	2 (0.8%)	1 (0.4%)	3 (0.7%)			
• Sometimes	7 (2.9%)	6 (2.1%)	18 (4.1%)			
• Almost never	19 (7.9%)	23 (8.2%)	92 (20.8%)			
• Never	210 (87.5%)	252 (89.4%)	329 (74.3%)			

Note. Data are presented as n (%). The total number of patients (denominator) varies for each TAI item due to missing responses. Statistical comparisons were made using the Chi-square test. Abbreviations: TAI, Test of Adherence to Inhalers; χ^2, Chi-square; df, degrees of freedom.

Supplementary Table S2 presents the complete, item-by-item data for the Test of Adherence to Inhalers (TAI), showing the distribution of all five response types across the 'Good', 'Insufficient', and 'Poor' clinical control groups. This table provides the detailed numerical data that is visually summarized in Supplementary Figure S5.

Supplementary Figure S6. Prevalence of Poor Clinical Control by Modifiable Factors and Clinical Severity Indicators

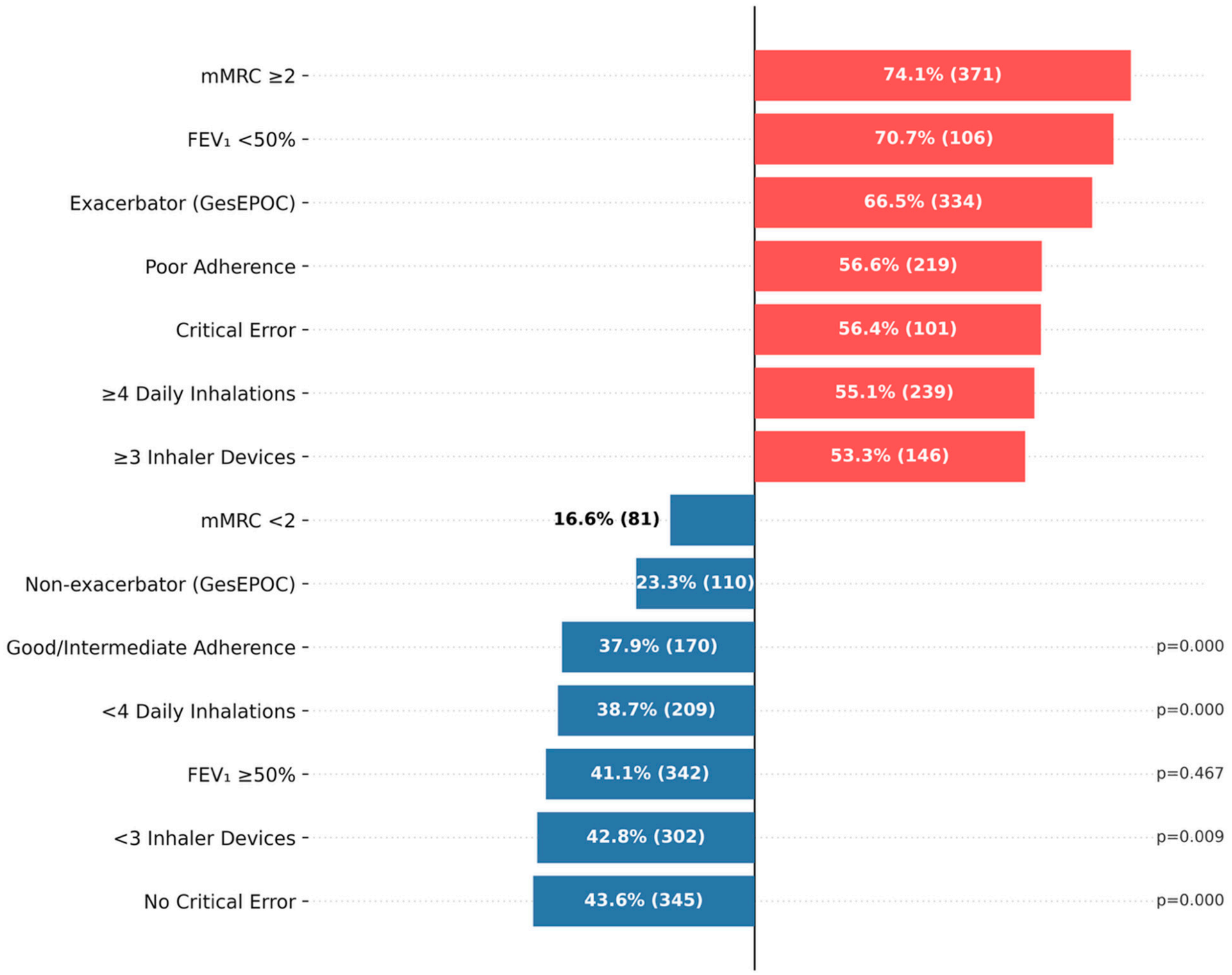

Note. The bar chart displays the prevalence of poor clinical control (RADAR score ≥4) across patient groups defined by modifiable treatment factors (adherence, number of inhaler devices, daily inhalations, and inhalation technique)

and clinical severity indicators (GesEPOC phenotype, mMRC dyspnea score, and FEV_1). For each comparison, the group with the higher prevalence of poor control is shown in red and the reference group in blue. Statistical comparisons are reported in the text.

The prevalence of poor clinical control was significantly associated with several modifiable patient behaviors and treatment factors (Supplementary Figure S6). Poor treatment adherence, the presence of critical inhalation errors, a higher number of daily inhalations (≥4 daily inhalations), and the use of three or more inhaler devices were all linked to a significantly higher likelihood of poor control compared to their respective reference groups (all p ≤ 0.004). For instance, the prevalence of poor control was 56.6% among patients with poor adherence, versus 37.9% in those with good or intermediate adherence (p < 0.001).

While modifiable factors were significant, the association was even stronger for clinical severity indicators. The prevalence of poor control was substantially higher in patients with an exacerbator phenotype (66.5% vs. 23.3%), significant dyspnea (74.1% vs. 16.6%), and severe airflow limitation (70.7% vs. 41.1%) compared to their less severe counterparts (all p < 0.001).

Supplementary Table S3. Detailed Characteristics of the Five Patient Phenotypes (N=452)

Variable	C0 — Multimorbid Exacerbators (n = 37)	C1 — Smoker-Dominant Frequent Exacerbators (n = 120)	C2 — High Inhalation Burden, Simple Devices (n = 127)	C3 — Device-Intense Severe COPD (n = 110)	C4 — Low Dyspnoea, Lower-Risk, Poor Adherence (n = 58)	*p*-value
Charlson Comorbidity Index†	4.0 [3.0–5.0]	2.0 [1.0–3.0]	2.0 [1.0–3.0]	3.0 [2.0–4.0]	2.0 [1.0–3.0]	< 0.001
Pack-years†	50.0 [34.0–65.0]	30.0 [20.0–40.0]	30.0 [20.0–45.0]	30.0 [23.0–40.0]	30.0 [21.0–39.0]	< 0.001
TAI-12 adherence†	48.0 [45.0–50.0]	44.0 [40.0–48.0]	45.0 [42.0–49.5]	44.0 [40.0–47.0]	42.0 [37.0–44.0]	< 0.001
Devices = 2‡	32 (86.5)	108 (90.8)	115 (92.7)	0 (0.0)	47 (81.0)	< 0.001
Devices = 3‡	3 (8.1)	10 (8.4)	0 (0.0)	110 (100.0)	8 (13.8)	< 0.001
Devices ≥4‡	2 (5.4)	1 (0.8)	9 (7.3)	0 (0.0)	3 (5.2)	< 0.001
Daily inhalations 2–3‡	37 (100.0)	111 (93.3)	0 (0.0)	16 (14.5)	45 (80.4)	< 0.001
Daily inhalations 4–6‡	0 (0.0)	8 (6.7)	105 (83.3)	66 (60.0)	8 (14.3)	< 0.001
Daily inhalations >6‡	0 (0.0)	0 (0.0)	21 (16.7)	28 (25.5)	3 (5.4)	< 0.001
FEV_1 ≥80%‡	5 (13.5)	19 (15.8)	8 (6.4)	11 (10.1)	10 (17.5)	0.032
FEV_1 50–79%‡	29 (78.4)	77 (64.2)	80 (64.0)	66 (60.6)	37 (64.9)	—
FEV_1 <50%‡	3 (8.1)	24 (20.0)	37 (29.6)	32 (29.4)	10 (17.5)	—
Smoker/ex-smoker‡	36 (97.3)	116 (96.7)	123 (96.9)	105 (95.5)	57 (100.0)	0.622
ICS use‡	21 (56.8)	56 (46.7)	66 (52.0)	67 (60.9)	23 (39.7)	0.041

Maintenance 3-drug therapy‡	17 (45.9)	41 (34.2)	51 (40.2)	60 (54.5)	18 (31.0)	0.017

Note. Data are presented as median [Interquartile Range] or n (%). †Median [IQR]. ‡n (%).

Supplementary Table S3 presents the detailed numerical data for the five patient clusters identified in the poorly controlled cohort (RADAR ≥4). This table provides the source data for Figure 5 in the main manuscript.
