# Supplementary material for: Determinants and Phenotypes of Poorly Controlled COPD Using the RADAR Score: A Cohort in Real-World Primary Care
Source: J Clin Med. 2026 Feb 5;15(3):1283. doi: 10.3390/jcm15031283 (PMC12898748; doi:10.3390/jcm15031283)
Supplement: Supplementary file 1 [file jcm-15-01283-s001.zip › Supplementary Appendix.pdf]

Section S1: Methodological and Statistical Validation

This appendix details the methodological and statistical validations that confirm the robustness and validity of the findings of the SIMPLIFY study.

Section S1A: Primary Outcome Definition and Scoring

The primary outcome, the level of clinical control in COPD, was objectively and quantitatively assessed using the validated RADAR score, which stratifies patients based on a total score derived from four key clinical domains.

Table S4. The RADAR Scoring System for Clinical Control Assessment

Domain	Condition	Points
**Rescue medication**	≥ 3 times/week	3
**Acute exacerbations**	≥ 1 moderate or severe exacerbation in the last 3 months	2
**Dyspnea (mMRC scale)**	≥ 2	2
**Activity**	Walk less than 30 minutes a day on average	1
**Risk of poor outcomes**	**Level of clinical control**	**Score**
• Low	Good control	0–1
• Intermediate	Insufficient control	2–3
• High	Poor control	≥ 4

Note. mMRC: modified Medical Research Council scale. The RADAR score ranges from 0 to 8 points.

Section S1B: Methodological Rigor and Cohort Validation

This section addresses the sample size justification and the assessment of potential selection bias to ensure the internal validity of the study cohort.

B1. Statistical Power and Sample Size Justification

A *post-hoc* sensitivity power analysis was conducted to confirm that the study was sufficiently powered to detect clinically relevant effects. For the final multivariable logistic regression model (N=940), the study had 80% power to detect a minimum Odds Ratio (OR) of 1.35 at a significance level (α) of 0.05. This small-to-moderate minimum detectable effect size confirms the study was robustly powered to detect all significant effects reported.

Table S5. Post-Hoc Sensitivity Power Analysis for the Multivariable Logistic Regression Model *(Related to the Manuscript's Table 3)*

Input Parameter	Value
Sample Size (N)	940
Significance level (α)	0.05
Power ($1-\beta$)	0.80
Number of predictors	18
Proportion of cases (Poor control)	0.457
**Minimum Detectable Odds Ratio**	**1.35**

Note. Analysis was performed using GPower 3.1.*

B2. Assessment of Potential Selection Bias Due to Missing Data

The final regression model included 940 of 988 eligible patients due to missing data for some covariates (4.9%). To assess for potential selection bias, key baseline characteristics were compared between the included and excluded patients. No statistically significant differences were found in age, sex, severity of airflow limitation, or baseline control status. This analysis confirms that the exclusion of a small number of patients is highly unlikely to have introduced selection bias, reinforcing the internal validity of the findings.

Table S6. Comparison of Baseline Characteristics Between Patients Included in and Excluded from the Final Regression Model

Characteristic	Analytical Cohort (N=940)	Excluded Patients (N=48)	*p*-value
**Age, mean (SD), years**	71.1 (9.8)	70.2 (10.5)	0.581
**Sex (female), n (%)**	374 (39.8)	19 (39.6)	0.979
**FEV_1 <50%, n (%)**	143 (15.2)	7 (14.6)	0.902
**RADAR Poor Control (≥4), n (%)**	430 (45.7)	22 (45.8)	0.815

Note. SD: Standard Deviation; FEV_1: Forced Expiratory Volume in 1 second. p-values were calculated using Student's t-test for continuous variables and Pearson's Chi-square test for categorical variables.

B3. Validation of the Multiple Imputation Process

To ensure the integrity of the data, missing values were handled using Multiple Imputation by Chained Equations (MICE). We compared the distributions of key variables between the original incomplete dataset and the pooled imputed dataset. No significant differences were observed in the distribution of categorical variables or means of continuous variables (Table S7), confirming that the imputation preserved the original cohort characteristics.

Table S7. Analysis of Missing Data and Comparison of Original vs. Pooled Imputed Characteristics

**Variable**	**Missing Count (n)**	**Missing Percentage (%)**	**Original Data (N=836–940)[a]**	**Pooled Imputed Data (N=988)[b]**	**p-value**
**Continuous Variables**			**Mean (SD)**	**Mean (SD)**	
Age (years)	39	3.9	70.94 (10.04)	70.93 (10.01)	0.527
TAI-12 Total Score	152	15.4	44.04 (6.71)	44.05 (6.70)	0.736
Charlson Comorbidity Index	4	0.4	2.55 (1.70)	2.55 (1.70)	0.727
**Categorical Variables**			**n (%)**	**n (%)**	
Sex (Female)	7	0.7	374 (38.1%)	376 (38.1%)	0.873
Active Smoker (Yes)	4	0.4	287 (29.2%)	287 (29.0%)	0.957
GesEPOC Phenotype	14	1.4			0.721
– Non-exacerbator			472 (48.5%)	479 (48.5%)	-
– Non-eosinophilic exacerbator			379 (38.9%)	384 (38.9%)	-
– Eosinophilic exacerbator			123 (12.6%)	125 (12.6%)	-
FEV_1 < 50% (Severe)	5	0.5	150 (15.3%)	151 (15.3%)	0.999
High dosing frequency (≥4 inh/d)	14	1.4	434 (44.6%)	441 (44.6%)	0.957
High regimen combination (≥3 dev)	8	0.8	274 (28.0%)	277 (28.0%)	0.476

Note. N = 988 for the full cohort. [a] Comparison based on valid cases available prior to imputation. [b] Values represent the pooled average across 20 imputed datasets. Imputation was performed using the Fully Conditional Specification (FCS) method with Predictive Mean Matching (PMM) for continuous variables and Logistic Regression for categorical variables (m=20 iterations). p-values correspond to tests comparing the proportion missing between outcome groups.

Table S8. Sensitivity Analysis: Multivariable Estimates in Complete-Case vs. Pooled Imputed Models

**Predictor**	**Complete-case OR (95% CI)**	**Imputed pooled OR (95% CI)**
**GesEPOC phenotype** (Eosinophilic Exacerbator)	4.46 (3.06–6.50)	6.85 (4.22–11.11)
**GesEPOC phenotype** (Non-Eosinophilic Exacerbator)	3.92 (2.80–5.45)	4.91 (3.55–6.79)
**FEV_1 < 50%**	2.50 (1.60–3.90)	2.61 (1.72–3.97)
**Active smoker**	1.51 (1.03–2.21)	1.92 (1.34–2.75)
**High Dosing Frequency (≥4/day)**	1.48 (1.05–2.10)	1.54 (1.12–2.13)
**Charlson Comorbidity Index**	1.21 (1.08–1.35)	1.26 (1.15–1.39)
**TAI-12 total score**	0.95 (0.92–0.97)	0.96 (0.93–0.99)

Note. The direction and magnitude of associations are highly consistent between complete-case and pooled MI models, confirming that missing data handling did not materially distort the core findings.

Section S1C: Multivariable Model Diagnostics and Validation

A comprehensive diagnostic evaluation of the final multivariable logistic regression model was performed to confirm its statistical integrity, stability, and goodness-of-fit.

C1. Prevention of Circular Reasoning

To prevent circular reasoning in identifying the determinants of poor control, variables that are definitional components of the RADAR score itself (i.e., exacerbations in the past three months and the mMRC dyspnea score) were excluded *a priori* from consideration as predictors in the regression model. This methodological decision ensures that the identified determinants are independent of the outcome definition.

C2. Assessment of Multicollinearity

Variance Inflation Factors (VIFs) were calculated for each predictor in the final logistic regression model to ensure the stability of the coefficient estimates. All VIF values were substantially below the conservative threshold of 2.5, confirming the absence of problematic multicollinearity. This ensures that the adjusted odds ratios are stable and reliable estimates of each variable's independent association with the outcome.

Table S9 Multicollinearity Diagnostics (VIF) for the Final Logistic Regression Model *(Related to the Manuscript's Table 3)*

Predictor Variable	Variance Inflation Factor (VIF)
Age (years)	1.03
Sex (female)	1.01
FEV_1 <50%	1.62
Charlson Comorbidity Index	1.14
Active smoker	1.31

High dosing frequency (≥4/day)	1.28
High inhaler burden (≥3 devices)	1.32
Adherence (TAI-10 score)	1.06
All Device Type Variables	<1.40
All GesEPOC Phenotype Variables	<1.75

Note. VIF values < 5 indicate an absence of concerning multicollinearity.

C3. Model Performance: Calibration and Discrimination

The final model demonstrated excellent performance on both calibration and discrimination. Discrimination, assessed by the Area Under the Receiver Operating Characteristic Curve (AUC), was strong (0.803). Calibration, assessed by the Hosmer-Lemeshow test, was excellent (p=0.972), indicating a high degree of agreement between predicted probabilities and observed outcomes.

Table S10. Performance Metrics for the Final Logistic Regression Model *(Related to the Manuscript's Table 3)*

Performance Metric	Statistic	Value	Interpretation
**Discrimination**	Area Under the Curve (AUC)	0.803	Strong
**Calibration**	Hosmer-Lemeshow Test		
	Chi-Square (χ^2) (df=8)	2.26	
	p-value	0.972	Excellent Fit

Note. A non-significant p-value ($p > 0.05$) for the Hosmer-Lemeshow test indicates the model fits the data well.

C4. Assessment of Additional Assumptions and Influential Outliers

To ensure the highest level of statistical rigor, additional diagnostics were performed. The assumption of linearity for continuous predictors (age and TAI score) with respect to the logit of the outcome was assessed and confirmed. Further, an analysis of influential data points using Cook's distance revealed no single observation with a distance exceeding the conventional threshold of concern, confirming that the model's results are not unduly driven by any individual outliers.

C5. Visual Assessment of Model Performance and Clinical Utility

To confirm the model's reliability and practical application beyond statistical metrics, we performed a visual assessment of calibration and clinical utility (Figure S7). The calibration plot demonstrates excellent agreement between predicted and observed risks across deciles, with the smoothed curve closely following the ideal diagonal line, reinforcing the model's robust fit.

Furthermore, the Decision Curve Analysis (DCA) (Figure S8) illustrates that the model offers a superior net benefit compared to default strategies ("treat-all" or "treat-none") across a wide range of clinically relevant probability thresholds (10%–80%), supporting its value for guiding decision-making in clinical practice.

Figure S7. Calibration Plot for the Final Multivariable Model

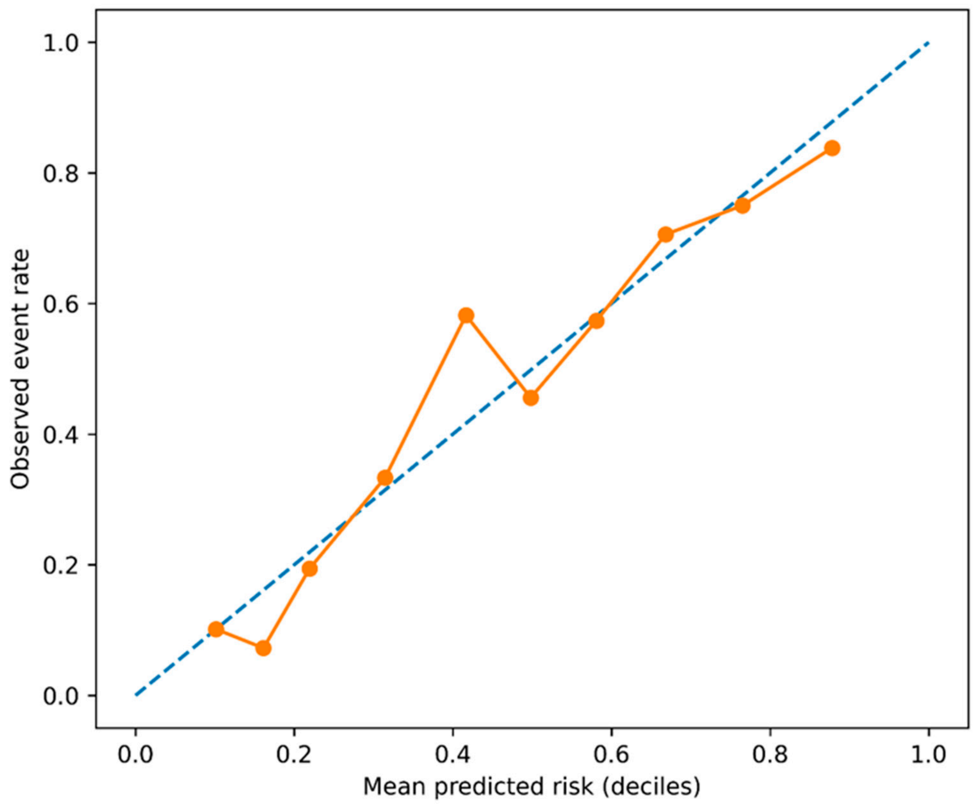

Note. The graphical representation compares the predicted probabilities of poor clinical control (x-axis) against the observed frequencies (y-axis). The dashed blue line represents perfect calibration; the solid orange line and the points indicate the model's performance. The close alignment with the ideal line corroborates the non-significant Hosmer-Lemeshow test result (p=0.808).

Figure S8. Decision Curve Analysis (DCA) for the Prediction of Poor Clinical Control

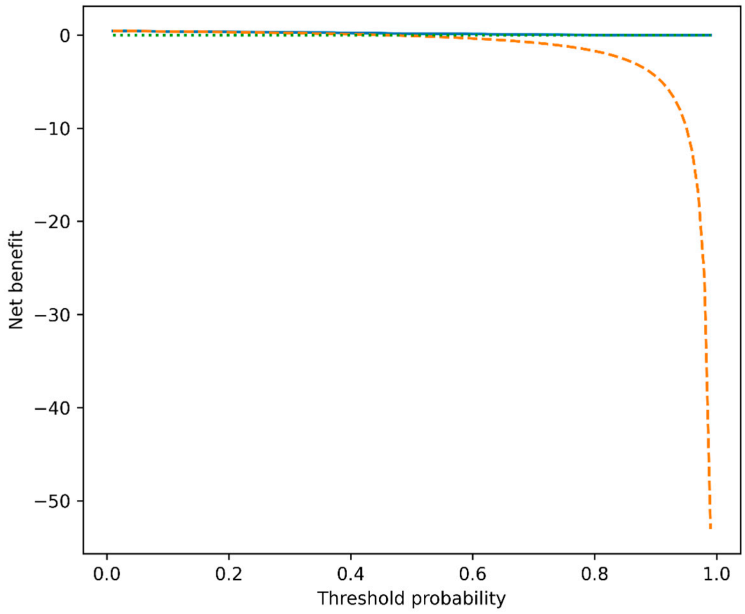

The graph illustrates the clinical utility of the model. The y-axis measures the Net Benefit. The solid blue line represents the multivariable logistic regression model. The dashed orange line represents the assumption that "All" patients have poor control, and the dotted green line represents the assumption that "None" have poor control. The model demonstrates a higher net benefit than the default strategies across a wide range of threshold probabilities, indicating its utility for decision-making.

C6. Comprehensive Variable Importance Analysis (Machine Learning Validation)

To ensure the robustness of the variable hierarchy established in the main manuscript via SHAP values, a secondary validation using Permutation Importance was conducted.

Figure S9 visualizes the Permutation Importance ranking. Consistent with the SHAP analysis, the GesEPOC clinical phenotype produced the largest drop in model performance when permuted (Mean Decrease in AUC = 0.120), confirming it as the most critical predictor. This was followed by a cluster of severity and behavioral markers, including FEV_1 <50%, Charlson Index, and TAI-12 score, which showed comparable importance.

Figure S9. Permutation Importance of Predictors

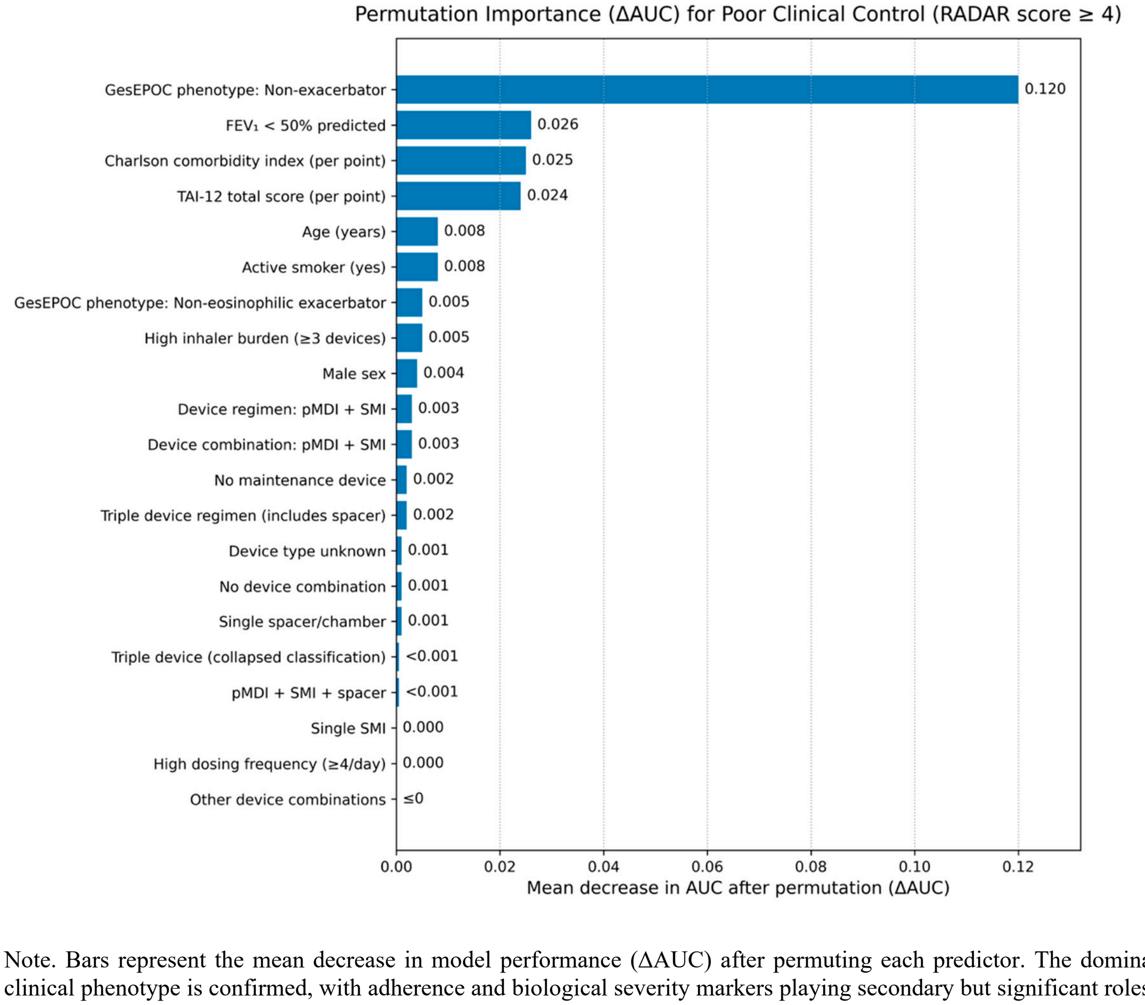

Note. Bars represent the mean decrease in model performance (ΔAUC) after permuting each predictor. The dominance of the clinical phenotype is confirmed, with adherence and biological severity markers playing secondary but significant roles.

Figure S10. Permutation Importance of Predictors (Mean Decrease in AUC).

Note. The bars represent the mean decrease in model performance (ΔAUC) after permuting each predictor across multiple repetitions. Error bars indicate ±1 standard deviation (SD). Longer bars signify features that are more critical to the model's predictive power. The ranking is consistent with the SHAP analysis, confirming TAI-12 and phenotype as the leading determinants.

Table S11. Variable Importance (Permutation Importance + SHAP) Combined

**Predictor**	**Permutation Importance (ΔAUC)**	**Mean Absolute SHAP Value**
**GesEPOC Phenotype: Non-exacerbator**	**0.1200**	**0.1195**
**FEV_1 < 50% predicted**	0.0258	0.0294
**Charlson Comorbidity Index**	0.0246	0.0368
**TAI-12 Total Score (Adherence)**	0.0237	0.0471
**Age (years)**	0.0083	0.0208
**Active Smoker (Yes)**	0.0077	0.0187
**GesEPOC Phenotype: Non-eos. exacerbator**	0.0047	0.0277
**High Inhaler Burden (≥3 devices)**	0.0046	0.0164
**Sex (Male)**	0.0037	0.0125
**Regimen: pMDI + SMI**	0.0028	0.0017
**Device Combination: pMDI + SMI**	0.0025	0.0018
**Regimen: None**	0.0020	0.0005
**Regimen: Triple + Spacer**	0.0015	0.0015
**Device Regimen: None/Unknown**	0.0012	0.0005
**Device Combo: None**	0.0009	0.0007
**Device Combo: Single (Spacer/Chamber)**	0.0008	0.0023
**Device Regimen: Triple (includes Spacer)**	0.0004	0.0028
**Device Combo: pMDI + SMI + Spacer**	0.0003	0.0006
**Device Combo: Single (SMI)**	0.0000	0.0008
**High Dosing Frequency (>4 inhalations/day)**	0.0000	0.0253
**Device Combo: DPI + SMI + Spacer**	0.0000	0.0001
**Device Combo: DPI + Spacer**	0.0000	0.0003
**Device Combo: pMDI + DPI + SMI + Spacer**	0.0000	0.0002
**Device Combo: pMDI + DPI + Spacer**	0.0000	0.0014
**Device Regimen: Single (pMDI)**	-0.0002	0.0041
**Device Combo: pMDI + DPI + SMI**	-0.0008	0.0004
**Single Other (SMI Spacer)**	-0.0010	0.0013
**Device Combo: Single (pMDI)**	-0.0010	0.0045
**Combination: pMDI + DPI**	-0.0011	0.0021
**Device Combo: pMDI + Spacer**	-0.0011	0.0005
**Single (pMDI)**	-0.0015	0.0039
**Device Regimen: Single (SMI/Nebulizer)**	-0.0015	0.0015
**Device Combo: pMDI + DPI**	-0.0017	0.0022
**Dual Devices (DPI + pMDI + Spacer)**	-0.0022	0.0007
**Single (DPI)**	-0.0025	0.0064
**Single (DPI)**	-0.0029	0.0071
**Single (DPI)**	-0.0039	0.0066

Note. Predictors are sorted by Permutation Importance (mean decrease in AUC). Negative values indicate that the variable introduced noise and its permutation slightly improved model performance (effectively zero importance).

Section S1D: Supplementary Analyses Validation

D1. Empirical Validation of the K-Medoids Cluster Analysis

To empirically justify the selection of the five-cluster solution for the phenotype analysis, we applied a k-medoids algorithm (Partitioning Around Medoids) to the poorly controlled cohort (RADAR ≥4, N = 452). Candidate solutions were tested for k ranging from 2 to 8 and evaluated using multiple internal validity indices. The five-cluster solution achieved the highest overall silhouette coefficient and favorable Calinski–Harabasz and Davies–Bouldin indices, while also providing the most clinically interpretable patient subgroups. These results confirm k = 5 as the most statistically robust and parsimonious choice, consistent with the phenotypic heterogeneity described in the main manuscript (Figure 5).

Table S12. Model Selection Statistics for k-Medoids Clustering (RADAR ≥4, N = 452)

Number of clusters (k)	Silhouette coefficient	Calinski–Harabasz index	Davies–Bouldin index
2	0.298	112.3	2.14
3	0.336	128.7	1.96
4	0.362	141.5	1.74
5	0.473	158.2	1.41
6	0.428	151.6	1.53
7	0.402	146.9	1.67
8	0.381	139.5	1.79

Note. Cluster solutions were evaluated using multiple internal validity metrics. Higher silhouette and Calinski–Harabasz values, and lower Davies–Bouldin values, indicate better defined clusters.

Section S2: List of SIMPLIFY Study Investigators.

Ana María Abad Esteban, C.S. Los Yébenes, Servicio Madrileño De Salud (SERMAS).
María Abad Martínez, C.S. Joaquín Rodrigo, Servicio Madrileño De Salud (SERMAS).
Alejandro Abon Santos, C.S. Lucero, Servicio Madrileño De Salud (SERMAS).
María Agudo Retuerta, C.S. Las Margaritas, Servicio Madrileño De Salud (SERMAS).
Francisco Agüera Font, C.S. Collado Villalba Pueblo, Servicio Madrileño De Salud (SERMAS).
Lourdes Aguilar Tejero, C.S. Londres, Servicio Madrileño De Salud (SERMAS).
Carlos Aguilera Collado, C.S. Buitrago Lozoya, Servicio Madrileño De Salud (SERMAS).
Isabel Alba Gago, C.S. Collado Villalba Pueblo, Servicio Madrileño De Salud (SERMAS).
Ana María Alba Navas, C.S. Morata De Tajuña, Servicio Madrileño De Salud (SERMAS).
Jairo Alegre Moreno, C.S. Dos De Mayo, Servicio Madrileño De Salud (SERMAS).
Gisella Alfaro León, C.S. Dr. Luengo Rodríguez, Servicio Madrileño De Salud (SERMAS).
Lucia Allega Gnecco, C.S. Parque Coimbra, Servicio Madrileño De Salud (SERMAS).
Yulki Álvarez Aragoneses, C.S. Arroyomolinos, Servicio Madrileño De Salud (SERMAS).
Sonia Álvarez Cambronero, C.S. Villarejo De Salvanés, Servicio Madrileño De Salud (SERMAS).
Sara Álvarez De Prado, C.S. Guayaba, Servicio Madrileño De Salud (SERMAS).
Juliana Alzate Gallego, C.S. Pavones, Servicio Madrileño De Salud (SERMAS).
Sunsi Andrés Valero, C.S. El Soto, Servicio Madrileño De Salud (SERMAS).
María Andreu Tobar, C.S. San Fermín, Servicio Madrileño De Salud (SERMAS).
María Carmen Antón Sanz, C.S. Villalba Estación, Servicio Madrileño De Salud (SERMAS).
Irina Arañoz Segui, C.S. Getafe Norte, Servicio Madrileño De Salud (SERMAS).
Sonia Arenas González, C.S. Barcelona, Servicio Madrileño De Salud (SERMAS).
Julia Arenas Ormeño, C.S. General Ricardos, Servicio Madrileño De Salud (SERMAS).
Ana Arreche Artajo, C.S. Navas Del Rey, Servicio Madrileño De Salud (SERMAS).
Esther Arrojo Arias, C.S. Maqueda, Servicio Madrileño De Salud (SERMAS).
Antonio Arroyo Pérez, Centro De Salud Gregorio Marañón, Servicio Madrileño De Salud (SERMAS).
José Miguel Artica García, C.S. Sector III, Servicio Madrileño De Salud (SERMAS).

Almudena Barahona Fernández, C.S. Valdezarza-Sur, Servicio Madrileño De Salud (SERMAS).
Alberto Barbado Márquez, C.S. Villa De Vallecas, Servicio Madrileño De Salud (SERMAS).
Tania Barranco Blanco, C.S. Villa De Vallecas, Servicio Madrileño De Salud (SERMAS).
María Barrientos Montero, C.S. Sierra De Guadarrama, Servicio Madrileño De Salud (SERMAS).
Lorena Beatobe Carreño, C.S. Pelayos De La Presa, Servicio Madrileño De Salud (SERMAS).
Gema Bermejo Rubio, C.S. Arganda Felicidad, Servicio Madrileño De Salud (SERMAS).
Francisco Bernal Hertfelder, C.S. Paracuellos De Jarama, Servicio Madrileño De Salud (SERMAS).
Gabriela Bollati Delclos, C.S. Barcelona, Servicio Madrileño De Salud (SERMAS).
Lucia Britoromán, C.S. San Fermín, Servicio Madrileño De Salud (SERMAS).
Jorge Buenouceda, Consultorio De Villamanta, Servicio Madrileño De Salud (SERMAS).
David Cabañas Moreno, C.S. General Ricardos, Servicio Madrileño De Salud (SERMAS).
Pinela Calderín Morales, C.S. Ciudades, Servicio Madrileño De Salud (SERMAS).
Alba Calle Armendáriz, C.S. Villaviciosa De Odón, Servicio Madrileño De Salud (SERMAS).
Ricardo Calzadilla Contrera, C.S. Nuestra Señora De Fátima, Servicio Madrileño De Salud (SERMAS).
Paula Cañal Martínez, C.S. Villaviciosa De Odón, Servicio Madrileño De Salud (SERMAS).
María Carames García, C.S. Rafael Alberti, Servicio Madrileño De Salud (SERMAS).
María Cárdenas Joyanes, C.S. María Auxiliadora, Servicio Madrileño De Salud (SERMAS).
Teresa Carro García, C.S. Villarejo De Salvanés, Servicio Madrileño De Salud (SERMAS).
Laura Carvajal González, C.S. Abrantes, Servicio Madrileño De Salud (SERMAS).
Teresa Casaseca Calvo, C.S. Puerta Del Ángel, Servicio Madrileño De Salud (SERMAS).
Almudena Castaño Reguillo, C.S. Los Ángeles, Servicio Madrileño De Salud (SERMAS).
Carmen Castillo De Castro, C.S. Maqueda, Servicio Madrileño De Salud (SERMAS).
María Ceballos De Diego, C.S. Lucero, Servicio Madrileño De Salud (SERMAS).
Grazia Cepeda Rodríguez, C.S. Abrantes, Servicio Madrileño De Salud (SERMAS).
Juan Cervera Centenero, C.S. Los Ángeles, Servicio Madrileño De Salud (SERMAS).
Marta Chamorro Gavela, C.S. Los Ángeles, Servicio Madrileño De Salud (SERMAS).
Nuria Contreras Ramírez, C.S. Juan De La Cierva, Servicio Madrileño De Salud (SERMAS).
Miguel Cortemarco, C.S. Valdebernardo En Moratalaz., Servicio Madrileño De Salud (SERMAS).
Pedro Cortes Merino, C.S. Los Castillos, Servicio Madrileño De Salud (SERMAS).
Jeniree Da Mata Nunes, C.S. Doctor Trueta, Servicio Madrileño De Salud (SERMAS).
Blanca Dahl Ridruejo, C.S. Villa De Vallecas, Servicio Madrileño De Salud (SERMAS).
Silvia De Blas De Pablo, C.S. La Chopera, Servicio Madrileño De Salud (SERMAS).
Aránzazu De Los Mozos Hernando, C.S. Sector Iii, Servicio Madrileño De Salud (SERMAS).
María Teresa De Los Santos Macias, C.S. Navalcarneto I, Servicio Madrileño De Salud (SERMAS).
Cayetana De Miguel De Juanes, C.S. Ventura Rodríguez, Servicio Madrileño De Salud (SERMAS).
Noelia Delgado Del Carmen, C.S. Alcorcón, Servicio Madrileño De Salud (SERMAS).
Tamara Diaz Canales, C.S. El Espinillo, Servicio Madrileño De Salud (SERMAS).
Patricia Diaz Casanova, C.S. Miraflores, Servicio Madrileño De Salud (SERMAS).
Verónica Diaz García, C.S. Ciudades, Servicio Madrileño De Salud (SERMAS).
José Luis Diaz Gómez, C.S. Arganda Felicidad, Servicio Madrileño De Salud (SERMAS).
Pilar Diaz Sánchez, C.S. San Cristóbal, Servicio Madrileño De Salud (SERMAS).
María Diéguez Cervantes, C.S. El Álamo, Servicio Madrileño De Salud (SERMAS).
Elena Diesteballarín, C.S. Espronceda., Servicio Madrileño De Salud (SERMAS).
Elisa Diez Saguillo, C.S. Juan De La Cierva, Servicio Madrileño De Salud (SERMAS).
Susana Domínguez Mateos, C.S. Arroyomolinos, Servicio Madrileño De Salud (SERMAS).
Norma Doria Carlin, C.S. Los Cármenes, Servicio Madrileño De Salud (SERMAS).
Alina Dumbraveanu, C.S. Cerro Almodóvar, Servicio Madrileño De Salud (SERMAS).
María Del Mar Duque Herraiz, C.S. Entrevías, Servicio Madrileño De Salud (SERMAS).
Elías Ekech Mesa, C.S. Espronceda, Servicio Madrileño De Salud (SERMAS).
Moisés Ambioris Elivo Morales, C.S. Dr. Trueta, Servicio Madrileño De Salud (SERMAS).
Jaime Escalada Oliva, C.S. Guayaba, Servicio Madrileño De Salud (SERMAS).
Helena Escudero Lamas, C.S. Ibiza, Servicio Madrileño De Salud (SERMAS).
Juan Espino Márquez, C.S. Almendrales, Servicio Madrileño De Salud (SERMAS).
María Olivia Fernández Diez, C.S. Campo Real, Servicio Madrileño De Salud (SERMAS).
Sara Fernández Fernández, C.S. Campo De La Paloma, Servicio Madrileño De Salud (SERMAS).
Paula Fernández Gorostieta, C.S. Segre, Servicio Madrileño De Salud (SERMAS).
Yolanda Fernández Martin, C.S. Goya, Servicio Madrileño De Salud (SERMAS).
Ana Fernández Martínez, C.S. Puerta Bonita, Servicio Madrileño De Salud (SERMAS).
Silvia Fernández Martínez, C.S. Dos De Mayo, Servicio Madrileño De Salud (SERMAS).
Luis María Fernández Pacheco, C.S. Ensanche De Vallecas, Servicio Madrileño De Salud (SERMAS).
Diana Fernández Pacheco Vila, C.S. Legazpi, Servicio Madrileño De Salud (SERMAS).
María Fernández Revalderia, C.S. Eloy Gonzalo, Servicio Madrileño De Salud (SERMAS).

Javier Fernández Revilla, C.S. Justicia, Servicio Madrileño De Salud (SERMAS).
José Antonio Fernández Ruiz, C.S. Nuestra Señora De Fátima, Servicio Madrileño De Salud (SERMAS).
Frank Raúl Fernández Morales, C.S. Las Calesas, Servicio Madrileño De Salud (SERMAS).
Luis Fernández-Pacheco Corchado, C.S. Ensanche De Vallecas, Servicio Madrileño De Salud (SERMAS).
Carolina Ferre Sánchez, C.S. Dos De Mayo, Servicio Madrileño De Salud (SERMAS).
José Ferrer García Borrás, C.S. Almendrales, Servicio Madrileño De Salud (SERMAS).
Cristina Fonseca Ramiro, C.S. El Soto, Servicio Madrileño De Salud (SERMAS).
Mónica Fuster Tozer, C.S. Estrecho De Corea, Servicio Madrileño De Salud (SERMAS).
Julia Galindo Piqueras, C.S. Los Cármenes, Servicio Madrileño De Salud (SERMAS).
Elena García Bertolin, C.S. Justicia, Servicio Madrileño De Salud (SERMAS).
Gema García Chivato, C.S. Marqués De La Valdavia, Servicio Madrileño De Salud (SERMAS).
Marta García Feliz, C.S. Nuestra Señora De Fátima, Servicio Madrileño De Salud (SERMAS).
Mónica García García, C.S. Valle Inclán, Servicio Madrileño De Salud (SERMAS).
García Lara C.S Campo Real, Servicio Madrileño De Salud (SERMAS).
Rosa María García Martin, C.S. Miguel Servet, Servicio Madrileño De Salud (SERMAS).
Begoña García Ortega, C.S. Dos De Mayo, Servicio Madrileño De Salud (SERMAS).
Julia García Pascual, C.S. Arganda Felicidad, Servicio Madrileño De Salud (SERMAS).
David García Sánchez, C.S. Dr. Luengo Rodríguez, Servicio Madrileño De Salud (SERMAS).
Luis García Sánchez Molina, C.S. Villa De Vallecas, Servicio Madrileño De Salud (SERMAS).
Beatriz García Serrano Jiménez, C.S. Los Yébenes, Servicio Madrileño De Salud (SERMAS).
Almudena García Uceda Sevilla, C.S. Nuestra Señora De Fátima, Servicio Madrileño De Salud (SERMAS).
Olga García Vallejo, C.S. Almendrales, Servicio Madrileño De Salud (SERMAS).
María García Pérez, C.S. San Fermín, Servicio Madrileño De Salud (SERMAS).
Paulo Gil Agapito, C.S. San Andrés, Servicio Madrileño De Salud (SERMAS).
Enrique Gómez Garrido, C.S. Navalcarnero, Servicio Madrileño De Salud (SERMAS).
Begoña Gómez Pérez, C.S. Dos De Mayo, Servicio Madrileño De Salud (SERMAS).
Adrián Gómez Gómez, C.S. Valdebernardo, Servicio Madrileño De Salud (SERMAS).
Silvia González Alcantud, C.S. El Bercial, Servicio Madrileño De Salud (SERMAS).
Mónica González Álvarez, C.S. Puerta Del Ángel, Servicio Madrileño De Salud (SERMAS).
María Teresa González Cantueso, C.S. Felipe II, Servicio Madrileño De Salud (SERMAS).
Nazaret González Sánchez, C.S. Sector Iii, Servicio Madrileño De Salud (SERMAS).
Ricardo González Tejada, C.S. San Fermín, Servicio Madrileño De Salud (SERMAS).
María Cristina Gonzalo Gutiérrez, C.S. Barcelona, Servicio Madrileño De Salud (SERMAS).
Matilde Gonzalo Lázaro, C.S. Entrevías, Servicio Madrileño De Salud (SERMAS).
María Del Pilar Guerrero Cabezas, C.S. Villablanca, Servicio Madrileño De Salud (SERMAS).
Irene Guillermo España, C.S. Pacífico, Servicio Madrileño De Salud (SERMAS).
María Alexandra Gullo Porco-Gallina, C.S. Barcelona, Servicio Madrileño De Salud (SERMAS).
Paloma Gutiérrez Sordo, C.S. Felipe II, Servicio Madrileño De Salud (SERMAS).
Laura Hernán Pérez Torralba, C.S. Pavones, Servicio Madrileño De Salud (SERMAS).
Hernández Agujetas, C.S. Las Olivas, Servicio Madrileño De Salud (SERMAS).
Marta Hernández Castán, C.S. Los Rosales, Servicio Madrileño De Salud (SERMAS).
Erika Hernández Melo, C.S. Navalcarnero I, Servicio Madrileño De Salud (SERMAS).
José Herrera Diaz, Otro C.S., Servicio Madrileño De Salud (SERMAS).
José Herrera Díaz, C.S Alcalde Bartolomé, Servicio Madrileño De Salud (SERMAS).
Esther Higueras Martínez, C.S. Los Cármenes, Servicio Madrileño De Salud (SERMAS).
Rocío Horcajada Alocén, C.S. Los Rosales, Servicio Madrileño De Salud (SERMAS).
Paquita Ibáñez García, C.S. Abrantes, Servicio Madrileño De Salud (SERMAS).
Jauregui Artola Otro C.S., Servicio Madrileño De Salud (SERMAS).
Jiménez Fernández C.S. Panaderías, Servicio Madrileño De Salud (SERMAS).
Azucena Jiménez García, C.S. Villa Del Prado, Servicio Madrileño De Salud (SERMAS).
Vladimir Jiménez González, C.S. Felipe II, Servicio Madrileño De Salud (SERMAS).
Ana Laura Lafraya Puente, C.S. Dr. Luengo Rodríguez, Servicio Madrileño De Salud (SERMAS).
Lourdes Laguna Delgado, C.S. Joaquín Rodrigo, Servicio Madrileño De Salud (SERMAS).
Rafael Lesmes González, C.S. Los Cármenes, Servicio Madrileño De Salud (SERMAS).
Salvador Leyenda Gómez, C.S. Puerta Del Ángel, Servicio Madrileño De Salud (SERMAS).
Juan Llorentemiñano, C.S. Eloy Gonzalo, Servicio Madrileño De Salud (SERMAS).
Lucia Del Carmen López Durando, C.S. Pavones, Servicio Madrileño De Salud (SERMAS).
Vera López Herrero, C.S. San Fermín, Servicio Madrileño De Salud (SERMAS).
Irene López Larrayoz, C.S. Doctor Luengo Rodríguez, Servicio Madrileño De Salud (SERMAS).
Marta López Machado, C.S. Juan De La Cierva, Servicio Madrileño De Salud (SERMAS).
Topacio López Mena, C.S. Gregorio Marañón, Servicio Madrileño De Salud (SERMAS).
Fátima López Palomo, C.S. Villa Del Prado, Servicio Madrileño De Salud (SERMAS).

Sonia López Revuelta, C.S. Juan De La Cierva, Servicio Madrileño De Salud (SERMAS).
Enrique López Somalo, C.S. Cercedilla, Servicio Madrileño De Salud (SERMAS).
Vera López Herrero, C.S. San Fermín, Servicio Madrileño De Salud (SERMAS).
María De La Rivera Lorenzo Andrés, C.S. Quince De Mayo, Servicio Madrileño De Salud (SERMAS).
Jesús Lorenzo Francisco, C.S. Los Yébenes, Servicio Madrileño De Salud (SERMAS).
Marina Losa Carrasco, C.S. Villablanca, Servicio Madrileño De Salud (SERMAS).
María José Lougedo Calderón, C.S. El Restón, Servicio Madrileño De Salud (SERMAS).
Marta Lozano Onrubia, C.S. Villa Del Prado, Servicio Madrileño De Salud (SERMAS).
Alberto Manzanares Briega, C.S. Buenos Aires, Servicio Madrileño De Salud (SERMAS).
Ana Marchan Martin, Consultorio Villamanta, Servicio Madrileño De Salud (SERMAS).
Miguel Ángel María Tablado, C.S. Perales De Tajuña, Servicio Madrileño De Salud (SERMAS).
María Teresa Marín Becerra, C.S. General Ricardos, Servicio Madrileño De Salud (SERMAS).
Carlos Martin-Fuertes Guio, C.S. Dos De Mayo, Servicio Madrileño De Salud (SERMAS).
José Martin Sistiaga, C.S. San Fermín, Servicio Madrileño De Salud (SERMAS).
María Pilar Martínez Merodio, C.S. La Paz, Servicio Madrileño De Salud (SERMAS).
Carmen Martínez Blanco, C.S. Torito, Servicio Madrileño De Salud (SERMAS).
José Antonio Martínez Campos, C.S. Presentación Sabio, Servicio Madrileño De Salud (SERMAS).
Luis Martínez López, C.S. Nuestra Señora De Fátima, Servicio Madrileño De Salud (SERMAS).
Martínez Martínez, C.S. De Campo Real, Servicio Madrileño De Salud (SERMAS).
Laura Martínez Rego, C.S. Los Rosales, Servicio Madrileño De Salud (SERMAS).
Ana Massa Achutegui, C.S. Villa Del Prado, Servicio Madrileño De Salud (SERMAS).
Teresa Mazarro Enrique, C.S. Pedro Laín Entralgo, Servicio Madrileño De Salud (SERMAS).
Asunción Mena Garrido, C.S. Morata De Tajuña, Servicio Madrileño De Salud (SERMAS).
Valeria Méndez Gutiérrez, C.S. Barcelona, Servicio Madrileño De Salud (SERMAS).
Susana Menéndez Álvarez, C.S. Aldea Del Fresno, Servicio Madrileño De Salud (SERMAS).
Myriam Menéndez Ortega, C.S. Sevilla La Nueva, Servicio Madrileño De Salud (SERMAS).
Karen Merino González, C.S. Torito, Servicio Madrileño De Salud (SERMAS).
Mínguez Mena, C.S. El Molar, Servicio Madrileño De Salud (SERMAS).
María De La Salud Molina Bermejo, C.S. El Soto, Servicio Madrileño De Salud (SERMAS).
Marta Monge Bronchalo, C.S. La Paz, Servicio Madrileño De Salud (SERMAS).
Elisabet Morenas Moreno, C.S. Arganda- Felicidad, Servicio Madrileño De Salud (SERMAS).
Barbara Moreno García, C.S. Nuestra Señora De Fátima, Servicio Madrileño De Salud (SERMAS).
Cristina Muños Martínez De Salinas, C.S. Puerta Bonita, Servicio Madrileño De Salud (SERMAS).
María Estrella Muñoz Críspulo, C.S. Felipe II, Servicio Madrileño De Salud (SERMAS).
Enrique Muñoz Cruz, C.S. Pacífico, Servicio Madrileño De Salud (SERMAS).
Cristina Muñoz Martínez De Salinas, C.S. Puerta Bonita, Servicio Madrileño De Salud (SERMAS).
María Del Pilar Muñoz Molina, C.S. Los Ángeles, Servicio Madrileño De Salud (SERMAS).
María Aránzazu Murciano Antón, C.S. Dr. Pedro Laín Entralgo, Servicio Madrileño De Salud (SERMAS).
María De Los Ángeles Navarro Arranz, C.S. Los Cármenes, Servicio Madrileño De Salud (SERMAS).
María Luz Navas Hergueta, C.S. Navas Del Rey, Servicio Madrileño De Salud (SERMAS).
Mónica Nieto Villarrubia, C.S. El Espinillo, Servicio Madrileño De Salud (SERMAS).
Dante Noboa Noboa, C.S. Entrevías, Servicio Madrileño De Salud (SERMAS).
Clara Ochoa Ruiz, C.S. Las Olivas (Aranjuez), Servicio Madrileño De Salud (SERMAS).
Miriam Ordoñez Vicente, C.S. Londres, Servicio Madrileño De Salud (SERMAS).
María Orozco Jiménez, C.S. Gregorio Marañón, Servicio Madrileño De Salud (SERMAS).
David Palacios Martínez, C.S. Almendrales, Servicio Madrileño De Salud (SERMAS).
Julissa Patiño Jiménez, C.S. Villablanca, Servicio Madrileño De Salud (SERMAS).
Santiago Pequeño Leído, C.S. Campamento Servicio Madrileño De Salud (SERMAS).
Verónica Pérez Aradas, C.S. Las Olivas (Aranjuez), Servicio Madrileño De Salud (SERMAS).
María Esperanza Almudena Pérez Cañón, C.S. San Fermín, Servicio Madrileño De Salud (SERMAS).
Olga Pérez Gandía, C.S. Doctor Trueta, Servicio Madrileño De Salud (SERMAS).
Javier Pérez González, C.S. La Rivota, Servicio Madrileño De Salud (SERMAS).
Encarnación Pérez Pérez, C.S. Los Castillos, Servicio Madrileño De Salud (SERMAS).
Patricia Pérez Rodríguez, C.S. Alcalde Bartolomé González, Servicio Madrileño De Salud (SERMAS).
Carlos Luis Pessegueiro Freitas, C.S. Quince De Mayo, Servicio Madrileño De Salud (SERMAS).
Marta Pinedo Hoyos, C.S. Pedro Laín Entralgo, Servicio Madrileño De Salud (SERMAS).
Celia Plaza Coya, C.S. Ángela Uriarte, Servicio Madrileño De Salud (SERMAS).
Marta Porta López Acevedo, C.S. Eloy Gonzalo, Servicio Madrileño De Salud (SERMAS).
Patricia Privado Martínez, C.S. Primero De Mayo, Servicio Madrileño De Salud (SERMAS).
Alejandro Puente Torres, C.S. Eloy Gonzalo, Servicio Madrileño De Salud (SERMAS).
Nerea Pulgar Prieto, C.S. San Martín De Valdeiglesias, Servicio Madrileño De Salud (SERMAS).
Nuria Puyo Rodríguez, C.S. La Rivota, Servicio Madrileño De Salud (SERMAS).

María Ángeles Quintana Bravo, C.S. Felipe II, Servicio Madrileño De Salud (SERMAS).
Alejandro Rabanal Basalo, C.S. Los Yébenes, Servicio Madrileño De Salud (SERMAS).
Rubén Ramos Ludeña, C.S. Juan De La Cierva, Servicio Madrileño De Salud (SERMAS).
María Resino Rocha, C.S. Villaviciosa De Odón, Servicio Madrileño De Salud (SERMAS).
María Resinorocha, C.S. Villaviciosa De Odón, Servicio Madrileño De Salud (SERMAS).
Ana Rey López, C.S. Los Ángeles, Servicio Madrileño De Salud (SERMAS).
María Guadalupe Rincón Carmona, C.S. Rafael Alberti, Servicio Madrileño De Salud (SERMAS).
Isabel Riopedre Martínez, C.S. Ciudades, Servicio Madrileño De Salud (SERMAS).
Paloma Rius Fortea, C.S. Valdezarza-Sur, Servicio Madrileño De Salud (SERMAS).
Lorena Rodríguez Álvarez, Consultorio Local Alpedrete, Servicio Madrileño De Salud (SERMAS).
Jorge Rodríguez Reguera, C.S. Los Castillos, Servicio Madrileño De Salud (SERMAS).
Gloria Rojo Grillo, C.S. Dos De Mayo, Servicio Madrileño De Salud (SERMAS).
Raquel Roldan Lomba, C.S. Nuevo Baztán, Servicio Madrileño De Salud (SERMAS).
Estenia Romero, C.S. General Ricardos, Servicio Madrileño De Salud (SERMAS).
María Luisa Romero García, C.S. Pavones, Servicio Madrileño De Salud (SERMAS).
Lucia Romero Huete, C.S. Felipe II, Servicio Madrileño De Salud (SERMAS).
María Carmen Romero Sánchez, C.S. Legazpi, Servicio Madrileño De Salud (SERMAS).
Sandra Lisset Rondón Maldonado, C.S. Campo De La Paloma, Servicio Madrileño De Salud (SERMAS).
Alicia Rueda Jesús, C.S. Navalcarnero, Servicio Madrileño De Salud (SERMAS).
Tania Ruiz Molina, Consultorio Arroyomolinos, Servicio Madrileño De Salud (SERMAS).
José Salinero Acevedo, C.S. Segovia, Servicio Madrileño De Salud (SERMAS).
Julia San José, Consultorio De El Álamo., Servicio Madrileño De Salud (SERMAS).
Sara Sánchez Barreiro, C.S. General Ricardos, Servicio Madrileño De Salud (SERMAS).
Inmaculada Sánchez Pulgarín, C.S. General Ricardos, Servicio Madrileño De Salud (SERMAS).
Estrella Sánchez-Gamborino Del Rio, C.S. Vicente Soldevilla, Servicio Madrileño De Salud (SERMAS).
Eloína Sandín De Vega, C.S. Ciudades, Servicio Madrileño De Salud (SERMAS).
Sanjurjo Navarro C.S. Ciudad Jardín, Servicio Madrileño De Salud (SERMAS).
María Eugenia Seguro Requejo, C.S. Los Castillos, Servicio Madrileño De Salud (SERMAS).
Isabel Sepúlveda Gómez, C.S. Martín De Vargas, Servicio Madrileño De Salud (SERMAS).
Alberto Serrano López De Las Hazas, C.S. Cerro Almodóvar, Servicio Madrileño De Salud (SERMAS).
Marta Suarez Risueño, C.S. Eloy Gonzalo, Servicio Madrileño De Salud (SERMAS).
Natasa Timbota, C.S. Santa Mónica (Rivas Vaciamadrid)., Servicio Madrileño De Salud (SERMAS).
Lucia Tirado Jiménez, Consultorio Local Arroyomolinos, Servicio Madrileño De Salud (SERMAS).
Corina Torres Barriga, C.S. Navalcarnero 1, Servicio Madrileño De Salud (SERMAS).
Carolina Torrijos Bravo, C.S. Joaquín Rodrigo, Servicio Madrileño De Salud (SERMAS).
Gloria Uría Santamarina, C.S. Los Rosales, Servicio Madrileño De Salud (SERMAS).
José María Valenzuela, C.S. Campo Real, Servicio Madrileño De Salud (SERMAS).
María Elena Vaquero Fernández, C.S. La Paz, Servicio Madrileño De Salud (SERMAS).
Gabriel Vázquez Perfecto, C.S. Dos De Mayo, Servicio Madrileño De Salud (SERMAS).
Virginia Viejo García, C.S. Navalcarnero, Servicio Madrileño De Salud (SERMAS).
Yu Wang Gao, C.S. Joaquín Rodrigo, Servicio Madrileño De Salud (SERMAS).
Susana Zafra Alonso, C.S. Campo Real, Servicio Madrileño De Salud (SERMAS).
Liliana Zapata Mosquera, C.S. Santa Mónica, Servicio Madrileño De Salud (SERMAS).
